# Development and validation of pretreatment nomogram for disease‐specific mortality in gastric cancer‐A competing risk analysis

**DOI:** 10.1002/cam4.4279

**Published:** 2021-10-10

**Authors:** Etsuro Bando, Xinge Ji, Michael W. Kattan, Maria Bencivenga, Giovanni de Manzoni, Masanori Terashima

**Affiliations:** ^1^ Division of Gastric Surgery Shizuoka Cancer Center Shizuoka Japan; ^2^ Department of Quantitative Health Sciences The Cleveland Clinic Cleveland OH USA; ^3^ Division of General and Upper Gastrointestinal Surgery Department of Surgery University of Verona Verona Italy

**Keywords:** competing risk, disease‐specific mortality, gastric cancer, nomogram

## Abstract

**Background:**

In several reports, gastric cancer nomograms for predicting overall or disease‐specific survival have been described. The American Joint Committee on Cancer (AJCC) introduced the attractiveness of disease‐specific mortality (DSM) as an endpoint of risk model. This study aimed to develop the first pretreatment gastric cancer nomogram for predicting DSM that considers competing risks (CRs).

**Methods:**

The prediction model was developed using data for 5231 gastric cancer patients. Fifteen prognosticators, which were registered at diagnosis, were evaluated. The nomogram for DSM was created as visualizations of the multivariable Fine and Gray regression model. An independent cohort for external validation consisted of 389 gastric cancer patients from a different institution. The performance of the model was assessed by discrimination (Harrell's concordance (C)‐index), calibration, and decision curve analysis. DSM and CRs were evaluated, paying special attention to host‐related factors such as age and Eastern Cooperative Oncology Group performance status (ECOG PS), by using Gray's univariable method.

**Results:**

Fourteen prognostic factors were selected to develop the nomogram. The new nomogram for DSM exhibited good discrimination. Its C‐index of 0.887 surpassed that of the American Joint Committee on Cancer (AJCC) clinical staging (0.794). The C‐index was 0.713 (AJCC, 0.582) for the external validation cohort. The nomogram showed good performance internally and externally, in the calibration and decision curve analysis. Host‐related factors including age and ECOG PS, were strongly correlated with competing risks.

**Conclusions:**

The newly developed nomogram accurately predicts DSM, which can be used for patient counseling in clinical practice.

## Graphical abstract

The newly developed gastric cancer nomogram for disease‐specific mortality accurately predicts treatment outcomes and thereby improve risk stratification and patient selection for routine clinical decision‐making and future clinical trials. This model will improve on the traditional categorical AJCC system in the future.

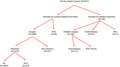

## 1 INTRODUCTION

The American Joint Committee on Cancer (AJCC) created a gastric cancer classification based on three criteria: the tumor depth (T), the degree of nodal involvement (N), and the distant metastases (M).^1^ This TNM system has been widely accepted for decades. However, the AJCC has increasingly recognized the need for more accurate risk prediction models that would incorporate additional tumor‐related or host‐related prognostic factors.^2^

Several reports have described gastric cancer nomograms after curative gastrectomy developed using postoperative pathological factors.^3^, ^4^, ^5^, ^6^ The selection of an initial therapy is primarily based on the clinical stage (cStage) at diagnosis.^7^, ^8^, ^9^ Furthermore, the use of neoadjuvant chemotherapy (NAC) is becoming increasingly common, especially in Western countries. Based on these circumstances, some studies reported a prognostic significance of pretreatment cStage.^10^, ^11^

Thus, a previous study developed a novel pretreatment gastric cancer nomogram (Figure S1) that predicts overall survival (OS) using 15 pretreatment variables, including non‐resection cases.^12^ The nomogram exhibited good discrimination and calibration, backed by internal and external validation cohort.

OS as an endpoint is consistent with the prior work of the AJCC. Clinicians are interested in disease‐specific survival (DSS) as well as OS. However, DSS considers surviving cases or those who died of other causes (competing risk [CR]) are censored. DSS reflects a hypothetical probability for patients: the chances of surviving their particular form of cancer assuming that they does not die of another cause first. According to some previous studies, DSS has certain biases.^13^, ^14^

The AJCC Precision Medicine Core later introduced the attractiveness of disease‐specific mortality (DSM).^2^ Gastric cancer patients may die of disease‐related death or from a CR. These two outcomes are mutually exclusive because a patient can never experience both events. DSM provides the probability of cancer‐specific death, and it can properly control CRs.^15^ Despite these situations, pretreatment gastric cancer risk models for DSM have not been reported, whereas nomograms for DSM have been developed for other malignancies.^16^, ^17^, ^18^

We aimed to develop the first gastric cancer pretreatment risk model using DSM as the endpoint by applying a proportional subdistribution hazards regression model,^19^ to improve patient counseling and assist ongoing efforts of the AJCC in developing the novel personalized staging.

## 2 MATERIALS AND METHODS

### 2.1 Cases and specimens

For the era of big data analysis,^19^ we collected data from/on 5231 patients (2002–2017) with histologically proven primary gastric cancer from the Shizuoka Cancer Center (Shizuoka, Japan) database. Patients with cancer of the remnant stomach or cancer that metastasized to the stomach from other organs were excluded from this study. We collected and registered data on tumor‐related variables (location, depth of invasion, number and anatomical extent of positive‐suspected regional nodes on CT, hepatic metastasis, peritoneal metastasis, other distant metastasis, macroscopic appearance, histologic differentiation, tumor size, serum concentration of carcinoembryonic antigen [CEA], and carbohydrate antigen 19–9 [CA19‐9]) and host‐related variables (age, sex, and Eastern Cooperative Oncology Group performance status [ECOG PS]). The determination of preoperative variables is presented (Supporting Methods and Table S1). This study was approved by the Institutional Review Board of Shizuoka Cancer Center (T29‐34–29–1, T30‐4–30–1).

A sample of 389 patients from the University of Verona (Verona, Italy) formed the external validation cohort. This external validation was approved by the Review Board of the Italian Research Group for Gastric Cancer.

### 2.2 Statistical analysis

Continuous variables were fit using a three‐knot cubic spline for potential nonlinear effects. The log‐rank was applied to evaluate differences of OS between groups.

The endpoint for developing the nomogram was DSM. The Gray method (for univariable nonparametric analysis) was used to assess the differences of DSM between groups. The Fine and Gray subdistribution model (for multivariable analysis)^20^ was employed to develop the DSM nomogram using all potential variables. Of the multiple prognostic variable combinations assessed, variables with the highest c‐index based on the step‐down reduction procedure were parsimoniously selected for the scale.^21^

We assessed the model performance internally and externally by examining discrimination (Harrell's concordance index (C‐index))^22^ and calibration plots. And we use decision curve analysis to plot the net benefit of model‐derived decisions.^23^ A *p*‐values <0.05 were deemed significant. R software (version 3.4.4) was used for all statistical analyses.

## 3 RESULTS

### 3.1 Patients’ demographics in the development cohort

**FIGURE 1:**
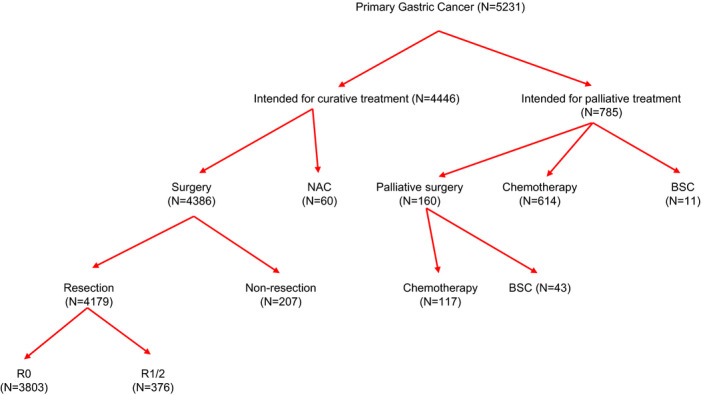
Summary of initial treatments. NAC, neoadjuvant chemotherapy; BSC, best supportive care

Clinicopathologic features of the development cohort are listed in Table 1. The initial treatment was determined on the basis of pretreatment cStage and the condition of patients. Figure 1 summarizes treatments administered to study participants. In total, 4446 patients (85.0%) were treated with curative intent, and 785 patients (15.0%) were treated with palliative intent.

**TABLE 1:** Descriptive statistics of pretreatment variables

A. Categorical variables	Development cohort		Validation cohort	
	N	(%)	N	(%)
Location				
Lower	1466	(28.0)	195	(50.1)
Middle	2182	(41.7)	101	(26.0)
Upper	1034	(19.8)	27	(6.9)
Entire	432	(8.3)	14	(3.6)
EGJ	117	(2.2)	52	(13.4)
Clinical T				
T1a	1101	(21.0)	1	(0.3)
T1b	1227	(23.5)	68	(17.5)
T2	532	(10.2)	64	(16.5)
T3	345	(6.6)	115	(29.6)
T4a	1809	(34.6)	108	(27.8)
T4b	217	(4.1)	33	(8.5)
Clinical N (anatomical location)				
N0	3398	(65.0)	159	(40.9)
N1	922	(17.6)	131	(33.7)
N2a	377	(7.2)	52	(13.4)
N2b	95	(1.8)	21	(5.4)
NM	439	(8.4)	26	(6.7)
Liver metastasis				
Negative	4889	(93.5)	377	(96.9)
Solitary	41	(0.8)	12	(3.1)
Multiple	301	(5.8)	0	(0.0)
Peritoneal dissemination				
Negative	4861	(92.9)	373	(95.9)
Positive	370	(7.1)	16	(4.1)
cM				
Negative	5103	(97.6)	382	(98.2)
Positive	128	(2.4)	7	(1.8)
Macroscopic type				
Type0	2670	(51.0)	35	(9.0)
Type1	169	(3.2)	29	(7.5)
Type2	830	(15.9)	98	(25.2)
Type3	1088	(20.8)	166	(42.7)
Type4	474	(9.1)	61	(15.7)
Histological differentiation				
G1	877	(16.8)	23	(5.9)
G2	1226	(23.4)	98	(25.2)
G3	3128	(59.8)	268	(68.9)
*Continued* Sex				
Female	1644	(31.4)	143	(36.8)
Male	3587	(68.6)	246	(63.2)
ECOG Performance Status				
0	4360	(83.3)	141	(36.2)
1	611	(11.7)	144	(37.0)
2	198	(3.8)	104	(26.7)
3 or 4	62	(1.2)	0	(0.0)

B. Continuous variables	median (range)	median (range)
Tumor size (mm)	40 (2–250)	40 (5–220)
Clinical N (number)	0 (0–42)	1 (0–13)
Age	67 (19–95)	68 (29–93)
Serum CEA (ng/ml)	2.3 (0.5–38640.0)	1.9 (0.5–429.0)
Serum CA19‐9 (U/ml)	8 (2–708200)	5 (2–2848)

Abbreviation: EGJ, esophago‐gastric junction tumor.

### 3.2 Survival outcomes

At the last follow‐up, 1,504 patients had died of gastric cancer and 350 had died of other causes. The other causes of death are summarized in Table S2.

**FIGURE 2:**
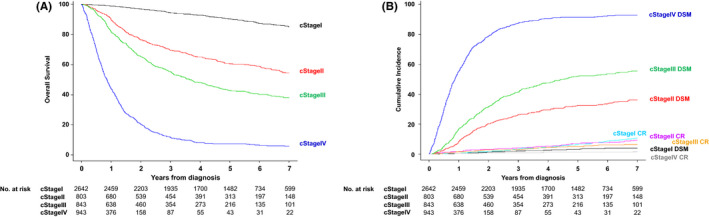
Treatment outcomes in development cohort according to the AJCC‐TNM staging. (A) Overall survival (OS). (B) Cumulative incidences of disease‐specific mortality (DSM) and competing risks (CR)

**FIGURE 3:**
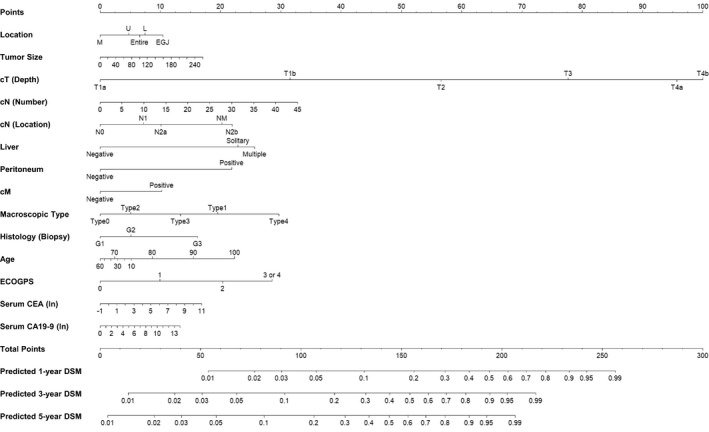
This nomogram allows the user to obtain the 1/3/5‐year probabilities of disease‐specific mortality (DSM). ln, natural logarithm

Figure 2A presents OS curves according to cStage. Figure 2B presents the cumulative incidences of DSM and CRs according to cStage. The Fine and Gray subdistribution model selected 14 variables to create the nomogram for DSM, except for sex (Table 2). A nomogram for DSM on the basis of this Fine and Gray model is presented in Figure 3. This nomogram allows the user to obtain the 1/3/5‐year probabilities of DSM. The regression equation for 5‐year DSM is shown in the Supporting Results.

**TABLE 2:** Multivariable Fine and Gray proportional subdistribution hazard models of pretreatment variables for disease‐specific mortality

Pretreatment variables		Chi‐Square	*p* value		
Location	Coefficients	11.59	0.021	HR	95% CI
Lower				1.25	1.08–1.45
Middle	−0.22570675			1	
Upper	−0.081076024			1.16	0.98–1.36
Entire	−0.026996459			1.22	0.99–1.51
EGJ	0.090486084			1.37	1.06–1.78
Tumor Size (mm)		2.45	0.118		
	0.001983954			1.09	0.98–1.21
cT		132.46	<0.001		
cT1a				0.06	0.03–0.10
cT1b	0.95211649			0.14	0.09–0.22
cT2	1.7092158			0.31	0.22–0.42
cT3	2.3470482			0.58	0.47–0.72
cT4a	2.8918279			1	
cT4b	3.0224211			1.14	0.94–1.38
cN (Number)		10.19	0.014		
	0.022027365			1.07	1.03–1.11
cN (Location)		28.33	<0.001		
cN0				1	
cN1	0.21855975			1.24	1.07–1.45
cN2a	0.30546798			1.36	1.11–1.64
cN2b	0.66233022			1.94	1.44–2.61
cNM	0.61197993			1.84	1.44–2.36
Liver Metastasis		72.64	<0.001		
Negative				1	
Solitary	0.69179943			2.00	1.40–2.86
Multiple	0.77610227			2.17	1.80–2.63
Peritoneum		62.36	<0.001		
Negative				1	
Positive	0.66090249			1.94	1.64–2.28
cM		5.74	0.017		
Negative				1	
Positive	0.3104542			1.36	1.06–1.76
Macroscopic Type		55.56	<0.001		
Type0				1	
Type1	0.59078268			1.81	1.18–2.76
Type2	0.15443309			1.17	0.83–1.64
Type3	0.40425807			1.50	1.08–2.09
Type4	0.89737586			2.45	1.71–3.53
Continued					
Histology		35.99	<0.001		
G1				0.61	0.50–0.75
G2	0.1561898			0.72	0.62–0.83
G3	0.48878957			1	
Age (Non‐Linear)		10.43	0.005		
				1.50	1.05–1.26
Age	−0.003401794				
Age−51	1.78469E−05				
Age−67	−4.16427E−05				
Age−79	2.37958E−05				
ECOG PS		46.83	<0.001		
0				1	
1	0.30036273			1.35	1.16–1.57
2	0.61478043			1.85	1.47–2.33
3 or 4	0.86264688			2.37	1.61–3.48
Serum CEA (ng/ml)		3.93	0.048		
	0.042532939			1.05	1.00–1.09
Serum CA19‐9 (U/ml)		4.23	0.040		
	0.028740838			1.15	1.00–1.10

Abbreviations: 95% CI, 95% confidence interval; HR, hazard ratio.

### 3.3 Internal validation

**FIGURE 4:**
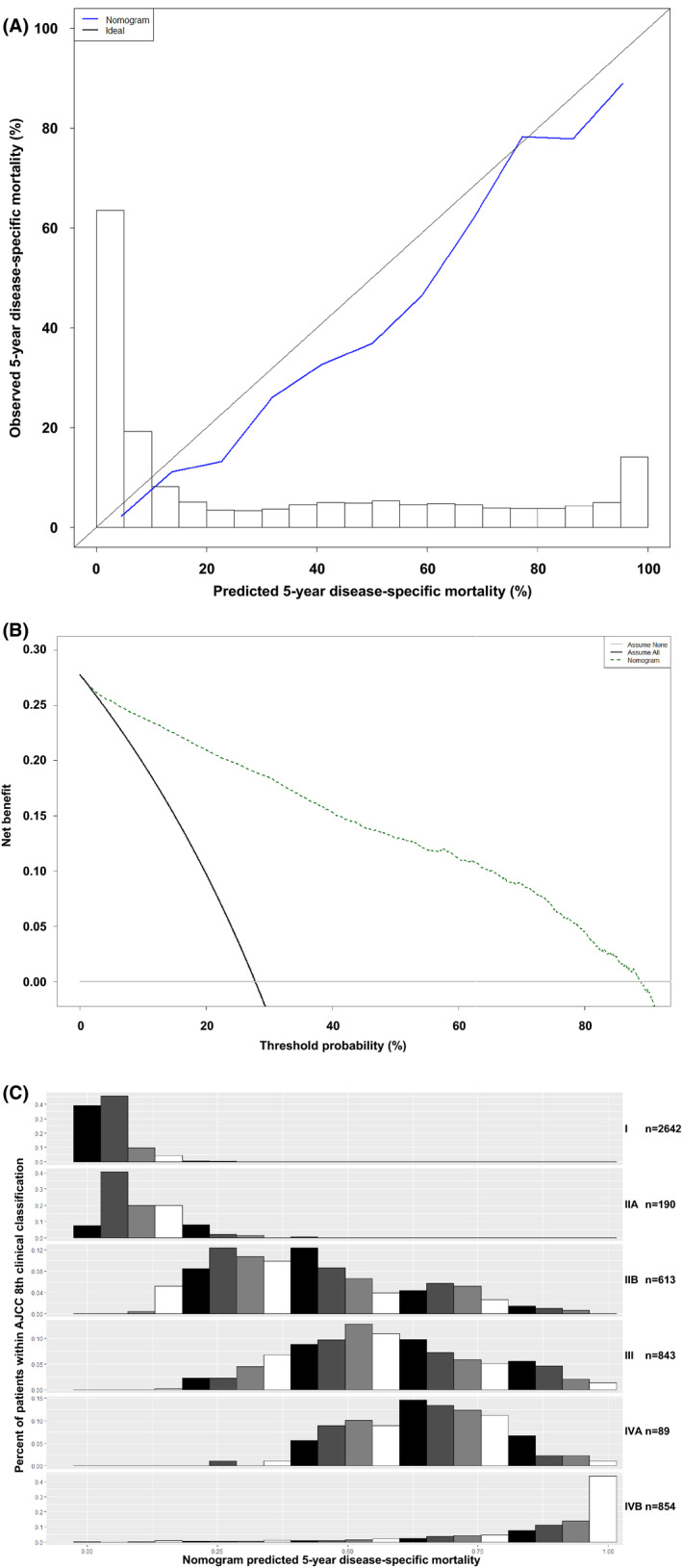
Internal validation of the nomogram for disease‐specific mortality (DSM) at the 5‐year endpoint. (A) Calibration plot. (B) Decision curve analysis. (C) Probabilities of nomogram predictions within each of the AJCC stage

The C‐index for the nomogram was 0.887, whereas that of the AJCC staging was 0.794. The calibration appeared to be accurate for the 5‐year prediction (Figure 4A). Compared with scenarios in which no prediction model was used for pretreatment decision‐making (i.e., assume all or assume none), the nomograms had a favorable net benefit across a wide range of decision threshold probabilities between 5‐year DSM probabilities of approximately 5 and 90% (Figure 4B). The nomogram‐predicted probabilities within each AJCC stage are presented in Figure 4C and were found to be heterogeneous within each stage, particularly in groups IIB, III, and IVA.

### 3.4 External validation

**FIGURE 5:**
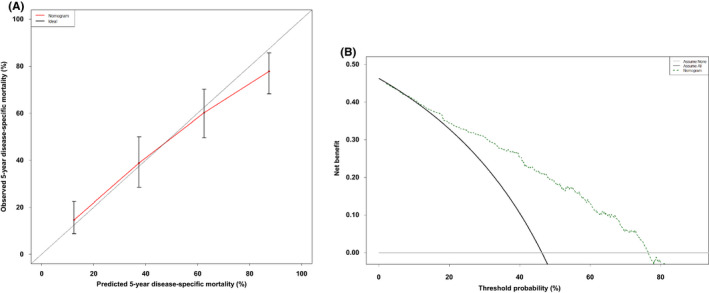
External validation of the nomogram for disease‐specific mortality (DSM) at the 5‐year endpoint. (A) Calibration plot. (B) Decision curve analysis

The University of Verona cohort was obtained from a surgical database, but it included 70 patients (18.0%) with cStage IV cancer. Clinicopathologic features of the external validation cohort are listed in Table 1. Thirty‐five patients (9.0%) received preoperative chemotherapy. The C‐index of the nomogram was 0.713, compared with 0.582 for AJCC clinical staging. The predicted and observed outcomes were in good agreement in the calibration plots (Figure 5A). In decision curve analysis, this model yielded a wide range of net benefits. The curve always exceeded the straight line of the “assume all”, ranging from 10% to 75% of threshold probabilities (Figure 5B).

### 3.5 Additional analyses

In addition, subset univariable survival analysis stratified by host‐related variables, including age and ECOG PS, which have been reported to be correlated with treatment outcomes or CRs,^12^, ^24^, ^25^ was performed. In all cases, age and ECOG PS had prognostic significance for OS (Figure S2A,B). A clear distribution was found concerning the cumulative incidence of CRs stratified by age (both *p* < 0.001; Figure S2C) as well as the cumulative incidence of DSM (both *p* < 0.001; Figure S2D). However, no significant differences were found regarding the cumulative incidence of DSM among age groups (*p* = 0.919 and *p* = 0.052) or the cumulative incidence of CR among all ECOG PS groups (*p* = 0.350 and *p* = 0.077). Furthermore, a stage‐specific subset prognostic evaluation stratified by age and ECOG PS was conducted (Figure S3–S6 and Supporting Results).

## 4 DISCUSSION

This study first developed a pretreatment gastric cancer nomogram for predicting disease‐specific mortality (DSM). The C‐index indicated that this nomogram had much better predictive ability than the current AJCC classification in a large cohort of patients.

For the era of tailored therapy, a previous study established a novel pretreatment nomogram predicting OS.^12^ This study chose DSM instead of DSS as an endpoint because it properly handles competing risks (CRs). Its clinical significance was demonstrated by the AJCC Precision Medicine Core.^2^

There were several strengths in this study. First, the development cohort included patients treated with both curative and non‐curative intents. Pretreatment clinical staging is vital for selecting therapeutic strategies, including surgery, NAC, chemoradiotherapy, or BSC. This nomogram should represent a good pretreatment tool for helping clinicians tailor treatment, replacing the current AJCC system. Second, this nomogram was successfully validated internally and externally using the Harrell C‐indices or calibration test as well as decision curve analysis. Thresholds in decision curve analyses are attractive for use in prospective trials. For example, if the inclusion criterion for a neoadjuvant clinical trial is a 5‐year DSM probability of more than 40%, a model‐based decision would have a clinical benefit of 0.15, indicating that the incidence of unnecessary treatment would be reduced by 15%.

In addition, more than half of the prognostic factors incorporated into the new nomogram are recommended for collection and registration by the AJCC^1^ and UICC.^26^ In Japan, the N category was based on the anatomical location of node metastasis^27^ until the AJCC published the 7th edition of the cancer classification.^28^ Some studies reported a correlation between survival and the anatomical location of pathologic lymph nodes,^29^, ^30^ but to date, no reports have discussed the prognostic value of the location of clinically positive nodes. A noteworthy point of our analysis was that metastasis suspected as N2b (Nos. 10, 11p/d, and 12a or Nos. 19, 20, and 111 in cases of esophagogastric junction tumors) had a strong negative prognostic impact, however N2a (Nos. 7, 8a, and 9) and N2b were defined in the same category of “N2” in previous Japanese gastric cancer classifications.^27^ Thus, information about both the number and location of suspicious nodes should be collected and registered. AJCC and UICC did not include macroscopic type as a recommended variable. However, the Japan Clinical Oncology Group performed a prospective clinical trial of perioperative chemotherapy targeting patients with diffuse‐type of macroscopic appearence^31^, ^32^ because of its poor treatment outcomes. We attempted to evaluate the prognostic significance of macroscopic type to assess its suitability for inclusion in the nomogram. In fact, a multivariable model identified macroscopic type as a significant factor for constructing new nomograms. In particular, Type 4 disease had strong negative prognostic accuracy. Thus, data on macroscopic type also should be collected and registered.

The AJCC staging of gastric cancer was based on OS.^11^, ^33^, ^34^, ^35^ It is possible that OS‐based staging prevents clinicians from selecting appropriate therapy. If the staging system or risk model predicts a 5‐year OS probability of 50%, normally intensive therapy, such as NAC or extended initial gastrectomy, will be planned. However, for patients aged 80 or older and those with an ECOG PS of 2 or higher, clinicians will avoid intensive therapy based on the possibility of therapy‐related death or future non‐cancer‐related death. Generally, standard treatment was established on the basis of randomized prospective trials with strict inclusion/exclusion criteria (e.g., age 75 or younger, ECOG PS of 0–1). Therefore, it is difficult to determine the treatment strategy for patients who deviate from these criteria.

In this study, we evaluated DSM, paying special attention to age and ECOG PS, which have been reported to be strongly correlated with competing events or treatment outcomes.^12^, ^25^ Age was a strong prognostic factor with a long horizontal axis in the OS nomogram (Figure S1) but a relatively weak prognostic factor with a short horizontal axis in the DSM nomogram (Figure 3). To help nomogram‐users understand, we performed univariable analysis stratified by age and ECOG PS (Figure S2–S6). This analysis was also applied to the external cohort. (Figure S7–S11). In the univariable analysis of OS (Figure S2–S11A only), age was a vital prognosticator in the stage‐specific subset evaluation both in the developing and validation cohorts, particularly in cStages I and II. In the univariable analysis for DSM using the Gray test (Figure S2–S11 B only), age was more strongly correlated with CR than DSM both in the developing and validation cohorts, particularly in cStages I–III. Conversely, ECOG PS had the same role for both OS and DSM with a moderate horizontal axis length in both nomograms. In the univariable analysis of OS (Figure S2‐S11C only), ECOG PS was also a significant prognostic factor in almost all stages, particularly in the developing cohort. In the univariable analysis of DSM (Figure S2–S11 D only), ECOG PS was more strongly correlated with CR than with DSM, particularly in the early cStages both in the developing and validation cohorts. Conversely, in the late cStages, ECOG PS was more strongly correlated with DSM, particularly in the developing cohort. These results indicated that patient age strongly correlated with CRs, whereas ECOG PS correlated with both CRs and DSM depending on tumor progression.

By using the multivariable Fine and Gray model to process such complicated prognostic factors, we developed a novel nomogram with much larger C‐index than that of the AJCC system. The difference in the C‐index is much larger than that in the OS analysis (Table S3). There can be a limitation when comparing rival prediction models when applied to separate data sets (endpoint).^36^ One possible reason is that the Fine and Gray model properly evaluates CRs by adding host‐related factors (age or ECOG PS). In other words, the Fine and Gray model accurately handles two different prognostic vectors.

At this moment, we have two nomograms. We believe that using separate pretreatment nomograms for OS and DSM should enhance their clinical value in the era of tailored therapy. To demonstrate the utility of these nomograms, two hypothetical cases are presented. A 50‐year‐old woman with cT3N+M0 poorly differentiated cancer (patient X, Table S4) had a 5‐year OS probability of 27% (OM (overall mortality) = 73%; Figure S12A) and a 5‐year DSM probability of 72% (Figure S12B). Intensive therapy should be planned because of the high DSM probability and low future CR probability. By contrast, an 86‐year‐old man with T1bN0M0 moderately differentiated cancer (patient Y, Table S4) had a 5‐year OS probability of 60% (OM = 40%; Figure S13A) and a 5‐year DSM probability of 9% (Figure S13B). Because of the high possibility of future CRs, low invasive therapy such as local resection or limited lymphadenectomy may be selected even if the guideline recommends standard gastrectomy. Adding the points of each variable together on the nomogram can be cumbersome. For this reason, we also developed risk calculator software (Figure S14A–B), similar to an online calculator on Cleveland Clinic website (http://riskcalc.org/).^37^

Despite several strengths, there were several limitations in this study. First, the nomogram was developed using data from retrospective databases. Second, the predictive accuracy of our nomogram is not perfect, and there is room for improvement. Our database does not provide other host‐related factors that are correlated with treatment outcomes, including nutrition status or comorbidity.^38^, ^39^ Third, patients in the external validation cohort were surgical curative‐intention cases whereas the developing cohort included palliative‐intention cases. One advantage of this model was that it can predict the DSM of non‐surgical and surgical cases; however, this biased selection of the external validation cohort might have affected the results. In fact, the C‐index of the external validation was not close to the value of the internal validation. Despite these limitations, our two models displayed the ability to stratify a population into individualized risk groups that can potentially reflect the risk–benefit balance of selecting therapy.

## 5 CONCLUSIONS

This study has developed the first pretreatment gastric cancer nomogram for predicting DSM on the basis of clinical and demographic risk factors using data obtained at diagnosis. In combination with the OS nomogram, the DSM nomogram has great utility for selecting appropriate initial therapy in consideration of the risk–benefit balance.

## CONFLICT OF INTEREST

The authors have no disclosures.

## AUTHOR CONTRIBUTIONS

Etsuro Bando: Conceptualization, formal analysis, investigation, methodology, and writing–original draft. Xinge Ji: Formal analysis, methodology, and writing–review and editing. Michael W. Kattan: Supervision, project administration, and writing–review and editing. Maria Bencivenga: Data curation and writing–review and editing. Giovanni de Manzoni: Resources and writing–review and editing. Masanori Terashima: Project administration and writing–original draft.

## ETHICAL APPROVAL STATEMENT

The study protocol has been approved by the Institutional Review Board of all participating institutes. The ethical guidelines of the World Medical Association Declaration of Helsinki—Ethical Principles for Medical Research Involving Human Subjects were fully conformed when conducting the present study.

## Supporting information

Supplementary Material

## Data Availability

The study data of development cohort are accessible.
