# Supplementary material for: Development and validation of pretreatment nomogram for disease‐specific mortality in gastric cancer‐A competing risk analysis
Source: Cancer Med. 2021 Oct 10;10(21):7561–71. doi: 10.1002/cam4.4279 (PMC8559461; doi:10.1002/cam4.4279)

**Supporting Information**

[**SUPPORTING METHODS** 2](#_Toc76479949)

[**REFERENCES** 4](#_Toc76479950)

[**SUPPORTING RESULTS** 5](#_Toc76479951)

[**Equation based on the nomogram for disease-specific mortality.** 5](#_Toc76479952)

[**Additional stage-specific analysis** 6](#_Toc76479953)

[**SUPPORTING TABLES** 8](#_Toc76479954)

[**TABLE S1** The definition of categories of pretreatment variables. 8](#_Toc76479955)

[**TABLE S2** Proportions of other causes of death (competing risks) in the developing cohort 9](#_Toc76479956)

[**TABLE S3** Comparison of Harrell's concordance index (C-index) between nomogram and the latest AJCC clinical staging based on disease-specific mortality (DSM) and overall survival (OS) 10](#_Toc76479957)

[**TABLE S4** Examples of nomogram use. Patient X: 50-year-old female patient with cT3 and nodal involvement (AJCC: cStageIII). Patient Y: 86-year-old male patient with cT1b and without nodal involvement (AJCC: cStageI). 11](#_Toc76479958)

[**SUPPORTING FIGURES** 12](#_Toc76479959)

[**FIGURE S1** This nomogram provides a method for calculating the 1-, 3-, and 5-year probabilities of overall survival (OS). 12](#_Toc76479960)

[**FIGURE S2** Survival outcomes in the development cohort (all patients). 13](#_Toc76479961)

[**FIGURE S3** Survival outcomes in patients with cStage I disease in the development cohort. 15](#_Toc76479962)

[**FIGURE S4** Survival outcomes in patients with cStage II disease in the development cohort. 17](#_Toc76479963)

[**FIGURE S5** Survival outcomes in patients with cStage III disease in the development cohort. 19](#_Toc76479964)

[**FIGURE S6** Survival outcomes in patients with cStage IV disease in the development cohort. 21](#_Toc76479965)

[**FIGURE S7** Survival outcomes in the external validation cohort (all patients). 23](#_Toc76479966)

[**FIGURE S8** Survival outcomes in patients with cStage I disease in the external validation cohort. 25](#_Toc76479967)

[**FIGURE S9** Survival outcomes in patients with cStage II disease in the external validation cohort. 27](#_Toc76479968)

[**FIGURE S10** Survival outcomes in patients with cStage III disease in the external validation cohort. 29](#_Toc76479969)

[**FIGURE S11** Survival outcomes in patients with cStage IV disease in the external validation cohort. 31](#_Toc76479970)

[**FIGURE S12** First example of nomogram use. Patient X: 50-year-old male with cT3 and nodal involvement (American Joint Committee on Cancer stage: cStage III). 33](#_Toc76479971)

[**FIGURE S13** Second example of nomogram use. Patient Y: 86-year-old male with cT1b but no nodal involvement (American Joint Committee on Cancer stage: cStage I). 35](#_Toc76479972)

[**FIGURE S14** Practical use of gastric cancer risk calculator. 37](#_Toc76479973)

# **SUPPORTING METHODS**

The definition of categories of pretreatment variables are summarized in Supporting Table1.

Eastern Cooperative Oncology Group Performance Status (ECOGPS)^1^ was described according to four score categories; 0, 1, 2, and “3 or 4”. Tumor location was subdivided into five categories: L; antrum, M; body, U; cardia, Entire; overlapping lesion, EGJ; esophagogastric junction tumor. Tumor length was measured by endoscopy or barium imaging.

Diagnosis of clinical tumor depth (cT) and clinically positive lymph nodes (cN) was determined according to a previously published report.^2^ cN-Number was treated as continuous variables, not converted to the current TNM category (N0/1/2/3a/3b). cN-Location was classified according to the following categories: N0; no positive nodes, N1; positive nodes in the perigastric area (No. 1, 2, 3a, 3b, 4sa, 4sb, 4d, 5 and 6, or No. 110 (paraesophageal nodes) if EGJ tumor), N2a; positive nodes in the suprapancreatic area (No. 7, 8a, and 9), N2b; positive nodes in the splenic hilum area, along the splenic artery, and in the hepatoduodenal ligament (No. 10, 11p, 11d, and 12a, or No. 19 (infradiaphragmatic nodes), No. 20 (esophageal hiatus), and No. 111 (supradiaphragmatic nodes) if EGJ tumor), and NM; positive nodes in intra-abdominal nonregional nodes (No. 12b/p, 13, 14a/v, 15, 16a/b, 17, and 18). This classification is referred to in the second English Edition of the Japanese Classification of Gastric Carcinoma and Japanese gastric cancer treatment guidelines.^3,4^ For cN2 classification only, we used our own classification. cN2a is the node within the area of limited D1+ lymphadenectomy, while cN2b is the node which does not dissected unless radical D2 dissection was performed.

Liver and peritoneum metastases were analyzed separately from cM due to their high frequency. We divided positive liver metastasis into solitary and multiple because solitary metastasis might be curable by liver resection.^5^ cM included metastasis to the lung, pleura, bone, central nervous system, skin, muscle, breast, spleen, adrenal glands, and extra-abdominal nodes. Macroscopic type was classified into Type 0 to Type 4 according to the Borrmann classification. Histology was classified into three groups: G1 (well differentiated type), G2 (moderately differentiated type), and G3 (poorly differentiated or undifferentiated type).

# **REFERENCES**

1. ECOG-ACRIN Cancer Research Group: ECOG Performance Status. <http://ecog-acrin.org/resources/ecog-performance-status>
2. Bando E, Makuuchi R, Tokunaga M, Tanizawa Y, Kawamura T, Terashima M. Impact of clinical tumor-node-metastasis staging on survival in gastric carcinoma patients receiving surgery. Gastric Cancer. 2017; 20: 448-456.
3. Japanese Gastric Cancer Association: Japanese Classification of Gastric Carcinoma (English ed 2). Gastric Cancer. 1998: 1: 10-24.
4. Japanese Gastric Cancer Association: Japanese gastric cancer treatment guidelines 2014 (ver 4). Gastric Cancer. 2017; 20: 1-19.
5. Markar SR, Mikhail S, Malietzis G, et al. Influence of Surgical Resection of Hepatic Metastases From Gastric Adenocarcinoma on Long-term Survival: Systematic Review and Pooled Analysis. Ann Surg. 2016; 263: 1092-1101.

# **SUPPORTING RESULTS**

## **Equation based on the nomogram for disease-specific mortality.**

Base is 0.9859575

- 0.081076024 * (Location = "U") - 0.22570675 * (Location = "M")

- 0.026996459 * (Location = "Entire") + 0.090486084 * (Location = "EGJ")

+ 0.0019839542 * Tumor Size + 0.95211649 * (cT = "T1b") + 1.7092158 * (cT = "T2") + 2.3470482 * (cT = "T3") + 2.8918279 * (cT = "T4a") + 3.0224211 * (cT = "T4b")

+ 0.022027365 * cN (Number) + 0.21855975 * (cN (Location) = "N1")

+ 0.30546798 * (cN (Location)= "N2a") + 0.66233022 * (cN (Location) = "N2b")

+ 0.61197993 * (cN (Location) = "NM") + 0.69179943 * (Liver = "Solitary")

+ 0.77610227 * (Liver = "Multiple")

+ 0.66090249 * (Peritoneum = "Positive") + 0.3104542 * (cM = "Positive")

+ 0.59078268 * (Macroscopic Type = "Type1") + 0.15443309 * (Macroscopic Type = "Type2") + 0.40425807 * (Macroscopic Type = "Type3")

+ 0.89737586 * (Macroscopic Type = "Type4")

+ 0.1561898 * (Histology = "G2") + 0.48878957 * (Histology = "G3")

- 0.0034017935 * Age + 0.000017846851 * max(Age - 51, 0)**3

- 0.000041642652 * max(Age - 67, 0)**3 + 0.000023795801 * max(Age - 79, 0)**3

+ 0.30036273 * (ECOGPS = "1") + 0.61478043 * (ECOGPS = "2")

+ 0.86264688 * (ECOGPS = "3 or 4")

+ 0.042532939 * SerumCEA.log

+ 0.028740838 * SerumCA19.log;

## **Additional stage-specific analysis**

A stage-specific subset survival analysis stratified by age and ECOG PS was conducted.

In cStage I, age and ECOG PS were the significant prognostic factors for OS (Figure S3A-B). The cumulative incidence of CR in patients aged 75 or older was significantly higher than that among patients aged 60–74 (*p* < 0.001), and the cumulative incidence of CR in patients aged 59 or younger was significantly lower than that among patients aged 60–74 (*p* < 0.001; Figure S3C). There was no significant difference in the cumulative incidence of DSM according to age (*p* = 0.983; Figure S3C). In the analysis stratified by ECOG PS, significant differences were found in the cumulative incidences of both DSM (*p* < 0.001) and CRs (*p* < 0.001) between PS0 and PS1–4 (Figure S3D).

In cStage II, age and ECOG PS were the significant prognostic factors for OS (Figure S4A-B). The cumulative incidence of CR in patients aged 75 or older was significantly higher than that in patients aged age 60–74 (*p* = 0.009), and the cumulative incidence of CRs in patients aged 59 or younger was significantly lower than that in patients aged 60–74 (*p* < 0.001; Figure S4C). The cumulative incidence of DSM in patients aged 75 or older was significantly higher than that in patients aged 60–74 (*p* < 0.001; Figure S4C). In the analysis stratified by ECOG PS, significant differences were found in the cumulative incidences of both DSM (*p* = 0.002) and CRs (*p* < 0.001) between PS0 and PS1–4 (Figure S4D).

In cStage III, older age and PS1–4 were the negative significant prognostic factors for OS (Figure S5A-B). The cumulative incidence of CRs in patients aged 75 or older was significantly higher than that in patients aged 60–74 (*p* = 0.016), and the cumulative incidence of CRs in patients aged 59 or younger was significantly lower than that in patients aged 60–74 (*p* = 0.004; Figure S5C). The cumulative incidence of DSM in patients aged 75 or older was significantly higher than that in patients aged 60–74 (*p* = 0.004; Figure S5C). In the analysis stratified by ECOG PS, a significant difference was found in the cumulative incidence of DSM between PS0 and PS1–4 (*p* < 0.001; Figure S5D). However, no significant difference was found in the cumulative incidence of CRs (*p* = 0.234) between PS0 and PS1–4 (see Supporting Fig. 5D).

In cStage IV, age was not a significant prognostic factor for OS, whereas ECOG PS was a significant factor (Figure S6A-B). A clear survival distribution was found among the three ECOG PS groups (Figure S6B). No significant differences were found in the incidences of DSM (*p* = 0.489) and CR (*p* = 0.124) stratified by age (Figure S6C). In the analysis stratified by ECOG PS, no significant difference was found in the cumulative incidence of CR (*p* = 0.365; Figure S6D). However, clear differences were found in the cumulative incidences of DSM between PS0 and PS1 and between PS1 and PS2–4 (both *p* < 0.001; Figure S6D).

| **SUPPORTING TABLES** | | | | | | |
| --- | --- | --- | --- | --- | --- | --- |
| **TABLE S1** The definition of categories of pretreatment variables. | | | | | | |
| **Pretreatment variables** |  |  |  |  |  |  |
| **Categorical variables** |  |  |  |  |  |  |
| Location | L | U | M | Entire | EGJ |  |
| cT (Depth) | T1a | T1b | T2 | T3 | T4a | T4b |
| cN (Location) | N0 | N1 | N2a | N2b | NM |  |
| Liver | Negative | Solitary | Multiple |  |  |  |
| Peritoneum | Negative | Positive |  |  |  |  |
| cM (Distant metastasis) | Negative | Positive |  |  |  |  |
| Macroscopic Type | Type0 | Type1 | Type2 | Type3 | Type4 |  |
| Sex | F | M |  |  |  |  |
| ECOGPS | 0 | 1 | 2 | 3 or 4 |  |  |
| Histology (Biopsy) | G1 | G2 | G3 |  |  |  |
| **Continuous variables** |  |  |  |  |  |  |
| Tumor Size (mm) |  |  |  |  |  |  |
| cN (Number) |  |  |  |  |  |  |
| Age |  |  |  |  |  |  |
| Serum CEA (ng/mL) |  |  |  |  |  |  |
| Serum CEA19-9 (U/mL) |  |  |  |  |  |  |
| Liver, liver metastasis; Peritoneum, peritoneal dissemination; cM, distant metastasis except metastasis in intra-abdominal nonregional lymph node, liver metastasis, and peritoneal dissemination; ECOGPS, Eastern Cooperative Oncology Group performance status; CEA, carcinoembryonic antigen; CA19-9, carbohydrate antigen 19-9. | | | | | | |

| **TABLE S2** Proportions of other causes of death (competing risks) in the developing cohort | | |
| --- | --- | --- |
| **Other cause of death** | **N** | **(%)** |
| Other malignant disease | 109 | (31.1) |
| Pulmonary disease | 85 | (24.3) |
| Stroke | 38 | (10.9) |
| Heart disease | 45 | (12.9) |
| Renal failure | 9 | (2.6) |
| Accident | 18 | (5.1) |
| Therapy related death | 26 | (7.4) |
| Others | 20 | (5.7) |

| **TABLE S3** Comparison of Harrell's concordance index (C-index) between nomogram and the latest AJCC clinical staging based on disease-specific mortality (DSM) and overall survival (OS) | | | | | |
| --- | --- | --- | --- | --- | --- |
|  |  | **DSM** | | **OS** | |
|  |  | C-index | 95% CI | C-index | 95% CI |
| Developing cohort | Nomogram | 0.887 | 0.881 - 0.894 | 0.855 | 0.848 - 0.863 |
|  | AJCC | 0.794 | 0.784 - 0.804 | 0.819 | 0.810 - 0.828 |
| External validation cohort | Nomogram | 0.713 | 0.680 - 0.746 | 0.714 | 0.681 - 0.746 |
|  | AJCC | 0.582 | 0.539 - 0.622 | 0.648 | 0.629 - 0.667 |
| AJCC, American Joint Committee on Cancer; 95% CI, 95% confidence interval. | | | | | |

| **TABLE S4** Examples of nomogram use. Patient X: 50-year-old female patient with cT3 and nodal involvement (AJCC: cStageIII). Patient Y: 86-year-old male patient with cT1b and without nodal involvement (AJCC: cStageI). | | |
| --- | --- | --- |
| **Pretreatment variables** | **Patient X** | **Patient Y** |
| Location | Entire | U |
| Tumor Size (mm) | 80 | 25 |
| cT (Depth) | T3 | T1b |
| cN (Number) | 9 | 0 |
| cN (Location) | N2a | N0 |
| Liver Metastasis | Negative | Negative |
| Peritoneum | Negative | Negative |
| cM | Negative | Negative |
| Macroscopic Type | Type4 | Type0 |
| Histology | G3 | G2 |
| Age | 50 | 86 |
| Sex | F | M |
| ECOG PS | 0 | 2 |
| Serum CEA (ng/ml) | 54.6 | 1.0 |
| Serum CA19-9 (U/ml) | 150 | 8 |
| AJCC, American Joint Committee on Cancer; cStage, clinical stage; cM excludes liver, peritoneum, intra-abdominal non regional metastasis, ECOG PS, Eastern Cooperative Oncology Group performance status. | | |

# **SUPPORTING FIGURES**

## **FIGURE S1** This nomogram provides a method for calculating the 1-, 3-, and 5-year probabilities of overall survival (OS).

Liver = liver metastasis. Peritoneum = peritoneal dissemination. cM = distant metastasis excluding metastasis to intra-abdominal non-regional lymph node, liver metastasis, and peritoneal dissemination. ECOG PS = ECOG performance status. CEA = carcinoembryonic antigen. CA19-9 = carbohydrate antigen 19-9.


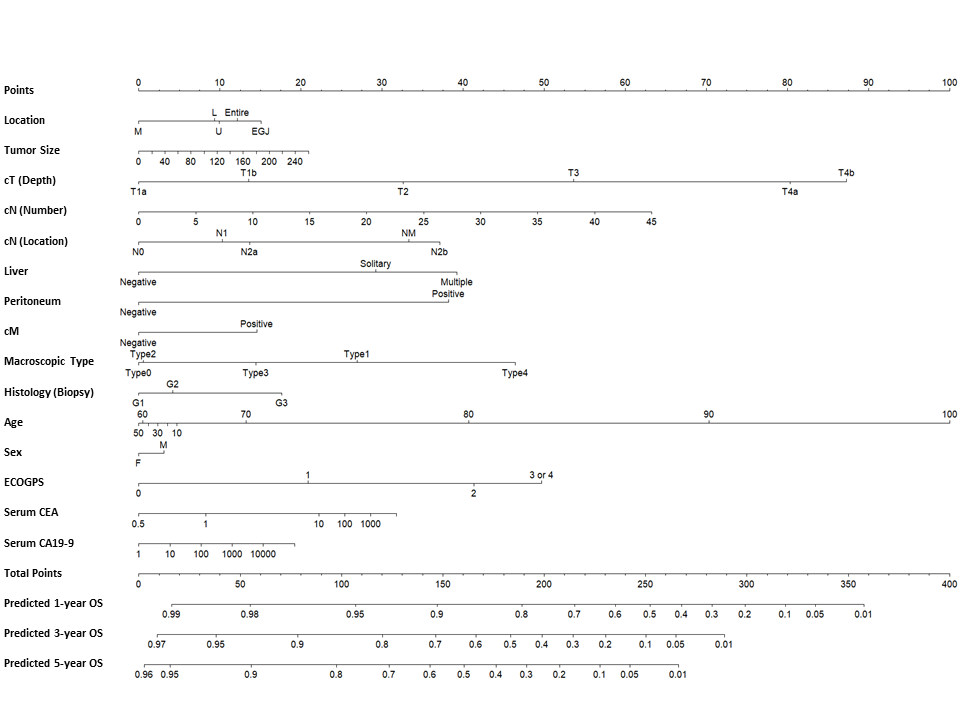


## **FIGURE S2** Survival outcomes in the development cohort (all patients).

(A) Overall survival stratified by age, (B) Overall survival stratified by the ECOG-PS.


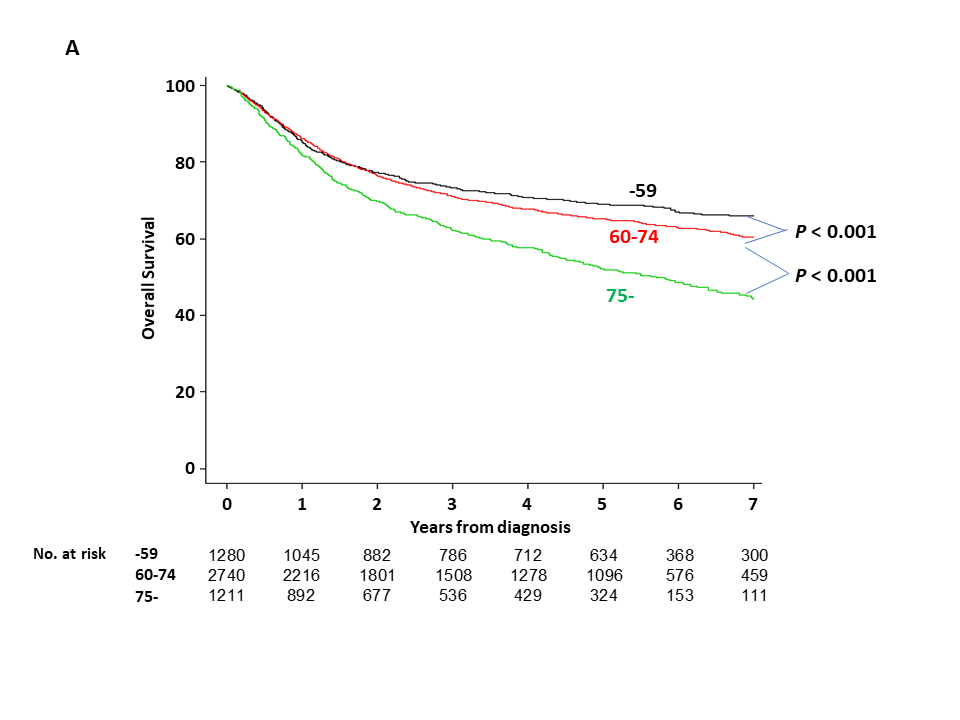


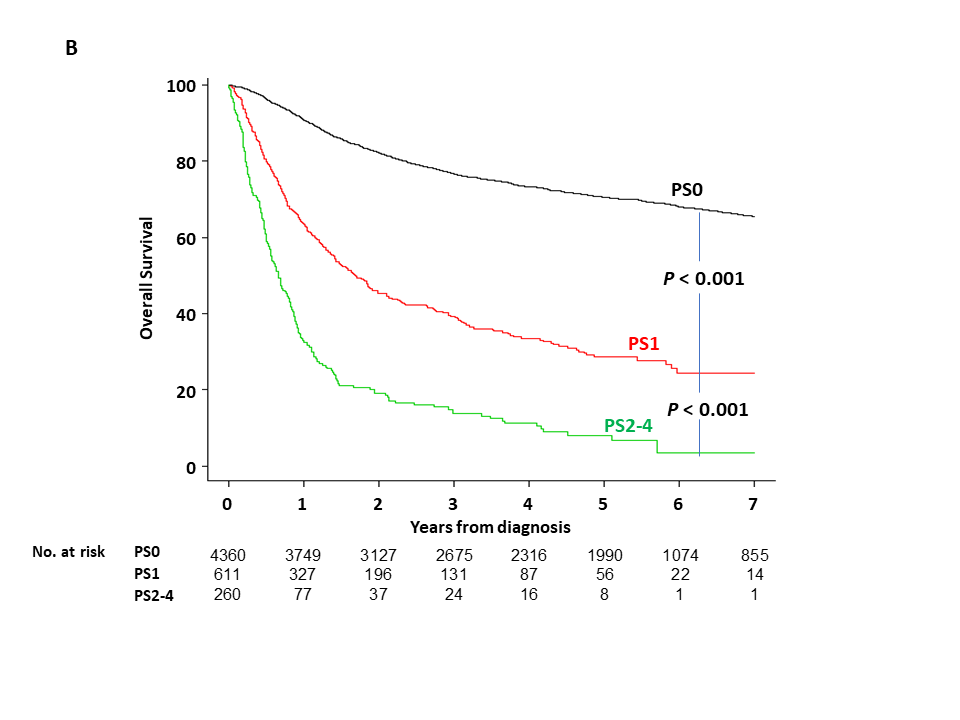


(C) Disease-specific mortality and competing risks stratified by age, (D) Disease-specific mortality and competing risks stratified by the ECOG-PS.


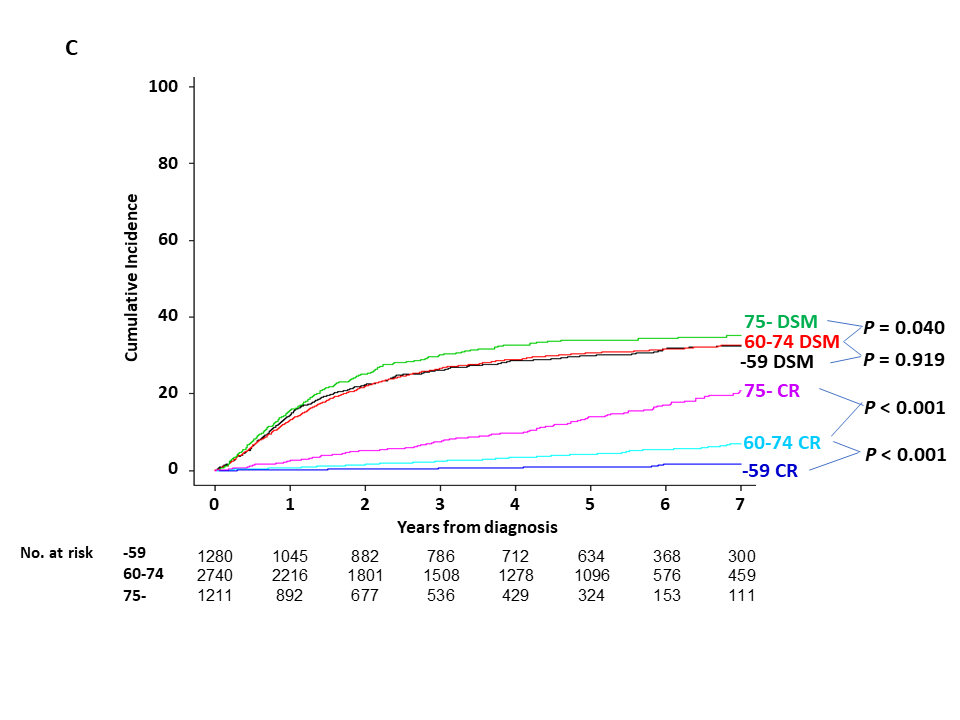


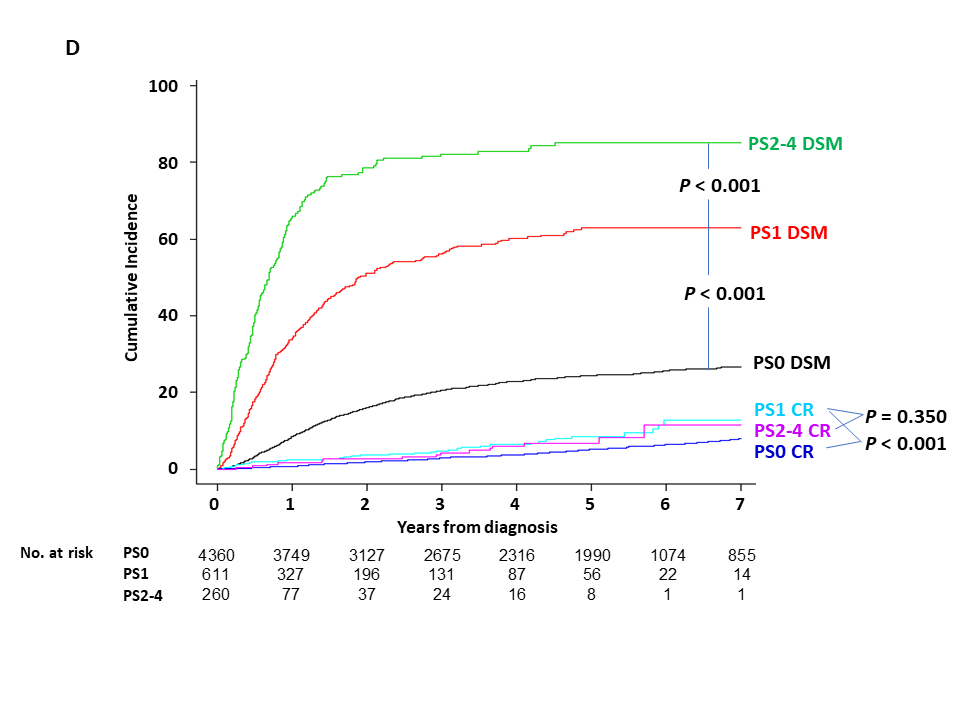


## **FIGURE S3** Survival outcomes in patients with cStage I disease in the development cohort.

(A) Overall survival stratified by age, (B) Overall survival stratified by the ECOG-PS.


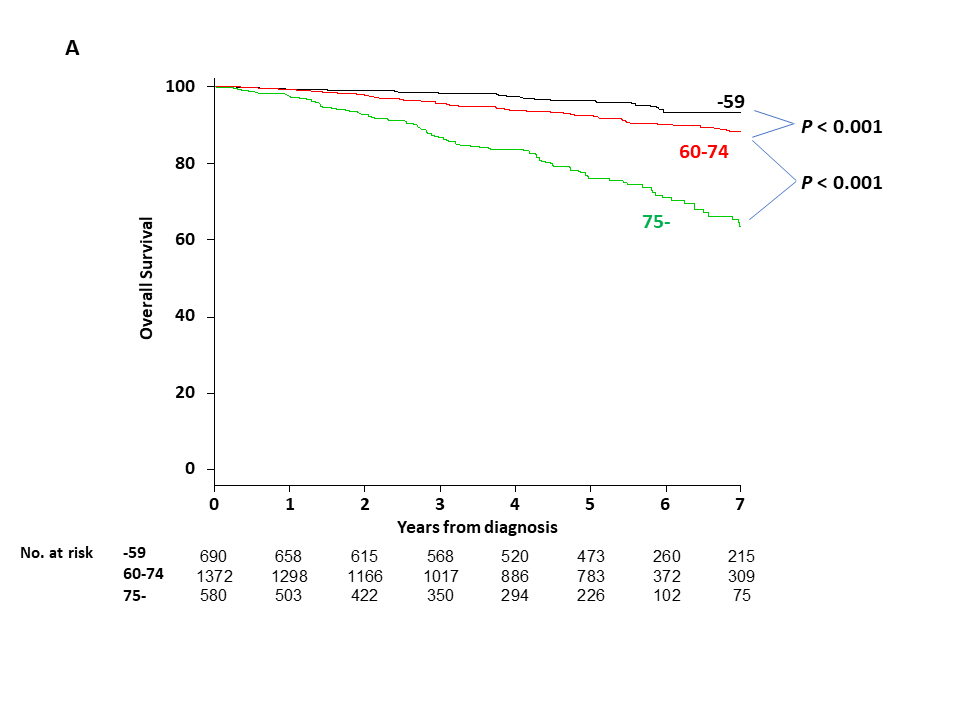


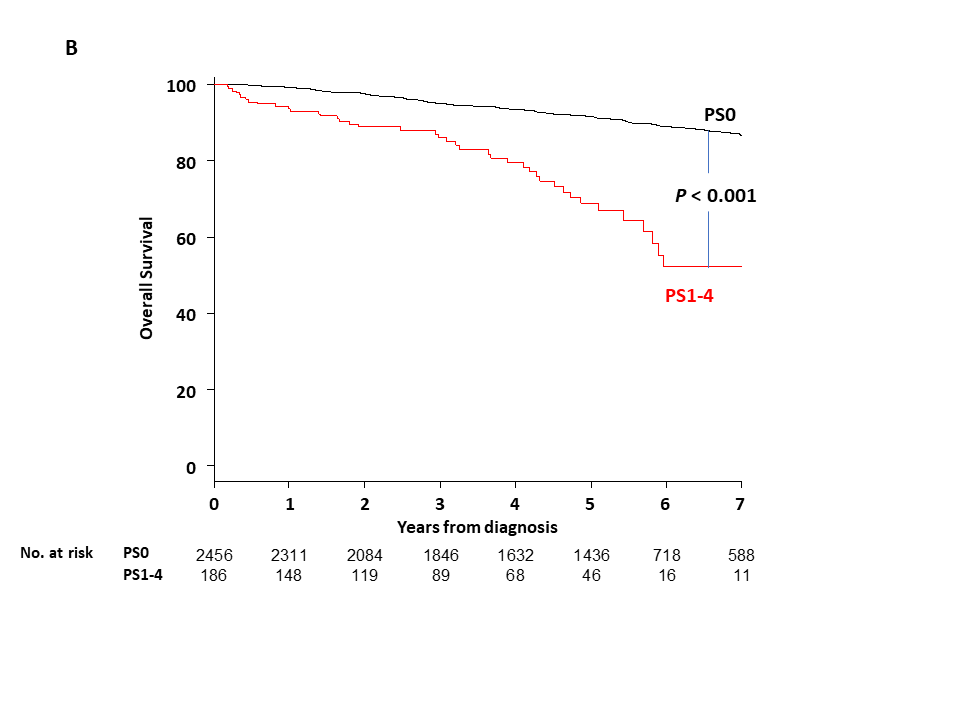


(C) Disease-specific mortality and competing risks stratified by age, (D) Disease-specific mortality and competing risks stratified by the ECOG-PS.


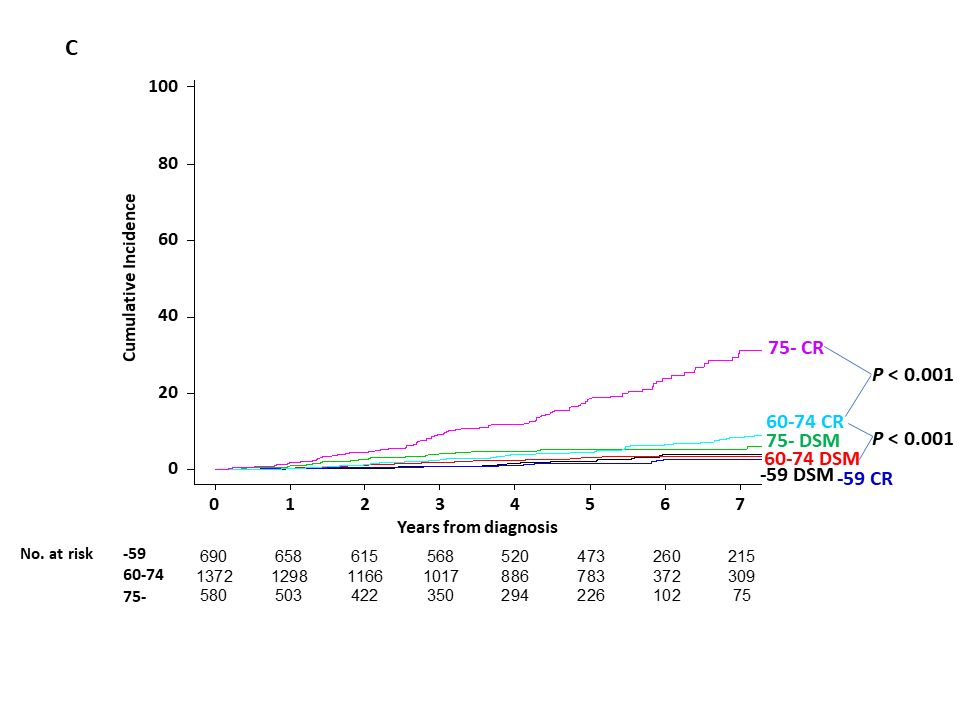


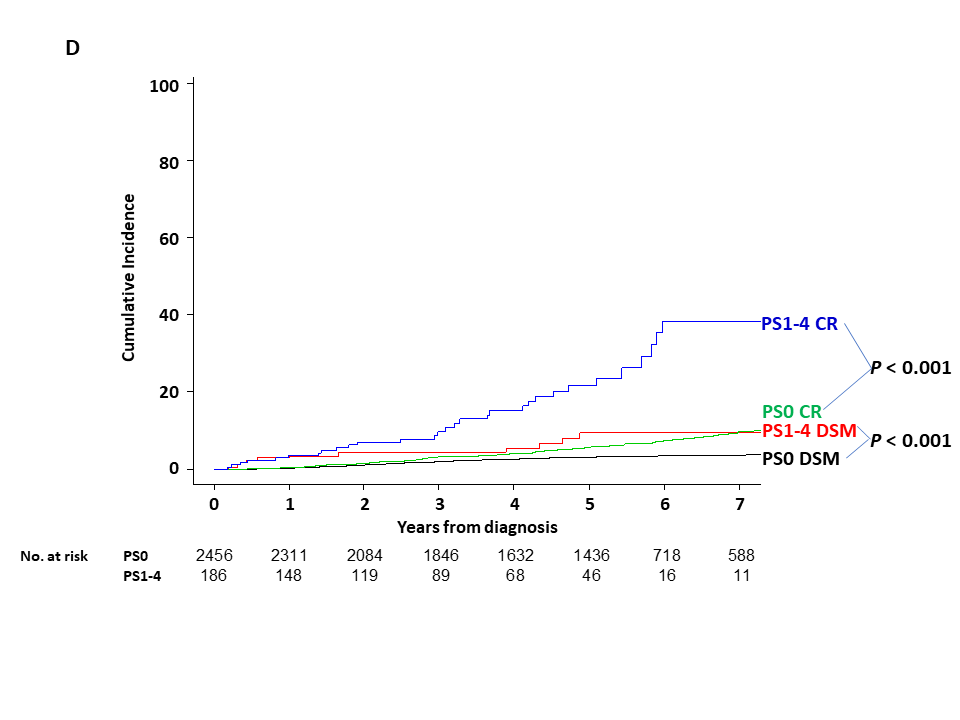


## **FIGURE S4** Survival outcomes in patients with cStage II disease in the development cohort.

(A) Overall survival stratified by age, (B) Overall survival stratified by the ECOG-PS.


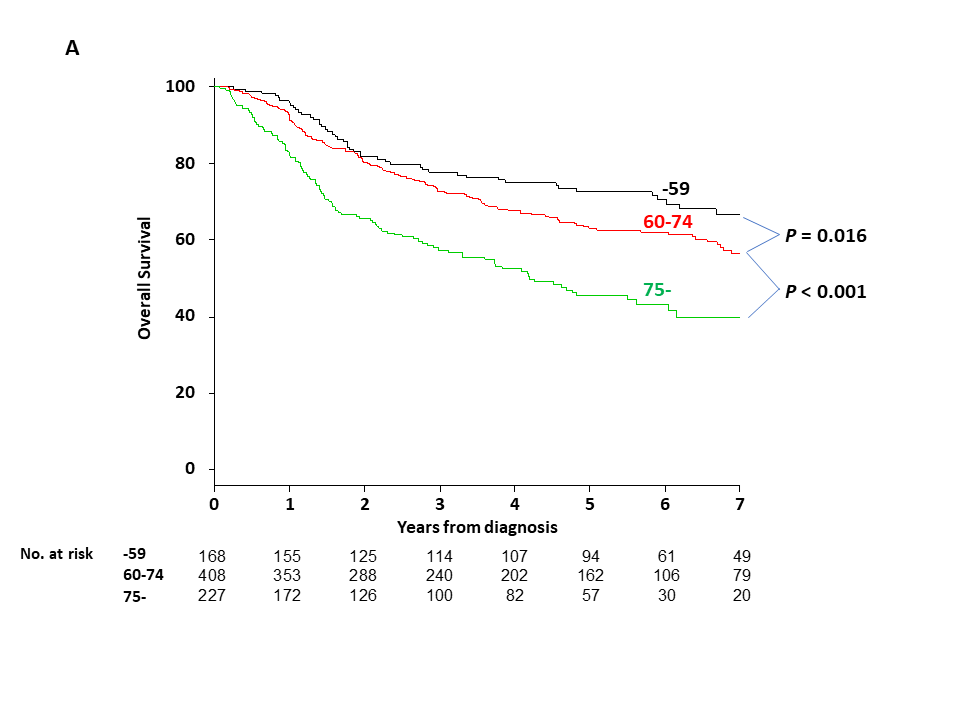


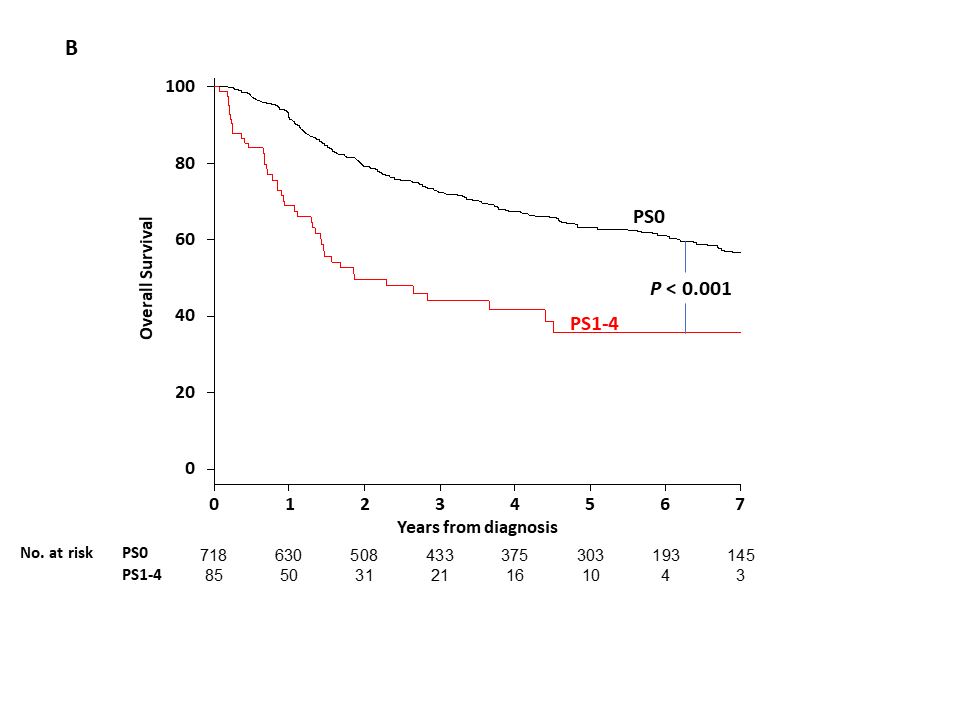


(C) Disease-specific mortality and competing risks stratified by age (D) Disease-specific mortality and competing risks stratified by the ECOG-PS.


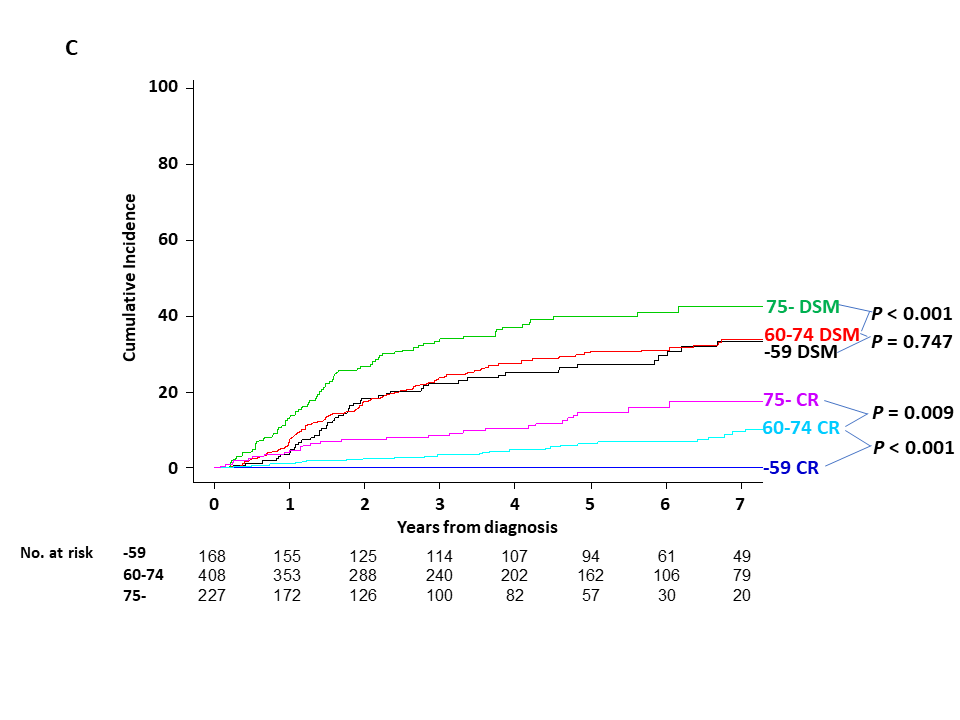


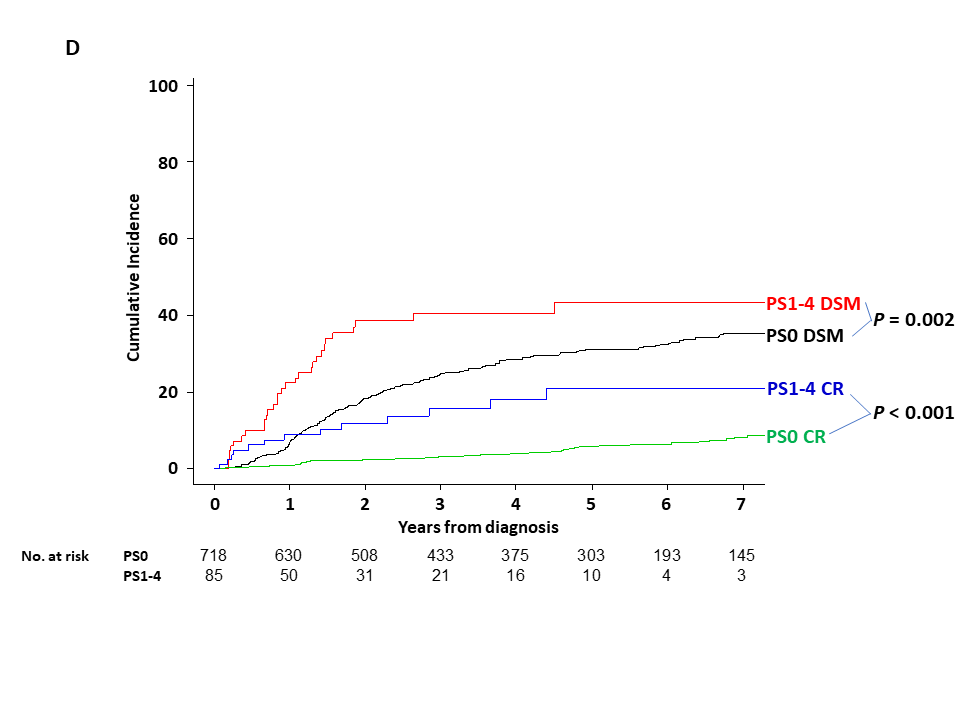


## **FIGURE S5** Survival outcomes in patients with cStage III disease in the development cohort.

(A) Overall survival stratified by age, (B) Overall survival stratified by the ECOG-PS.


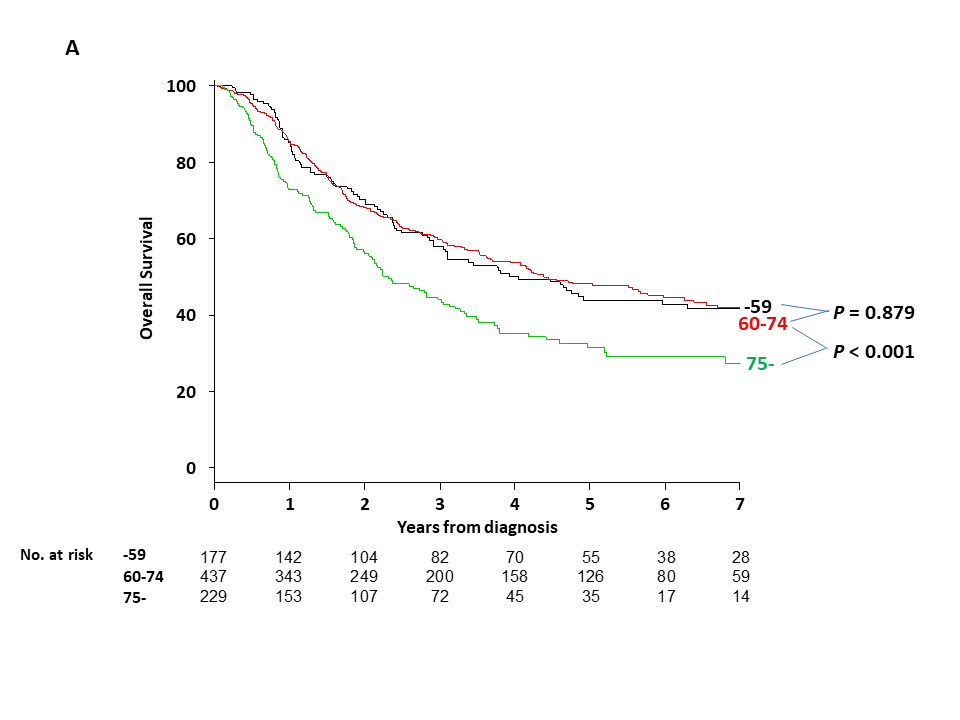


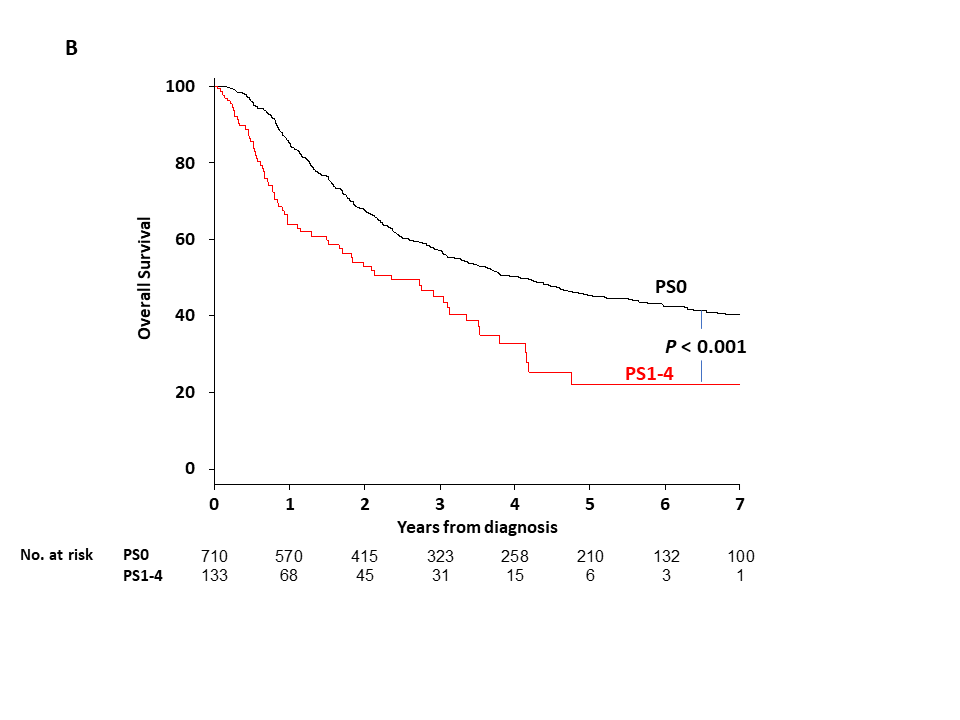


(C) Disease-specific mortality and competing risks stratified by age, (D) Disease-specific mortality and competing risks stratified by the ECOG-PS.


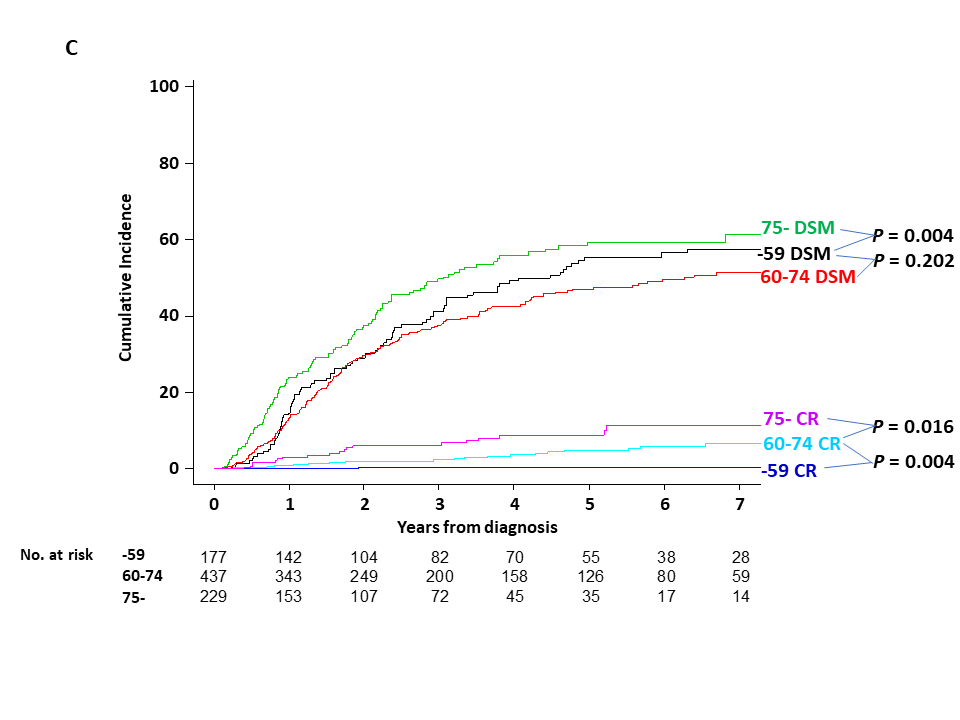

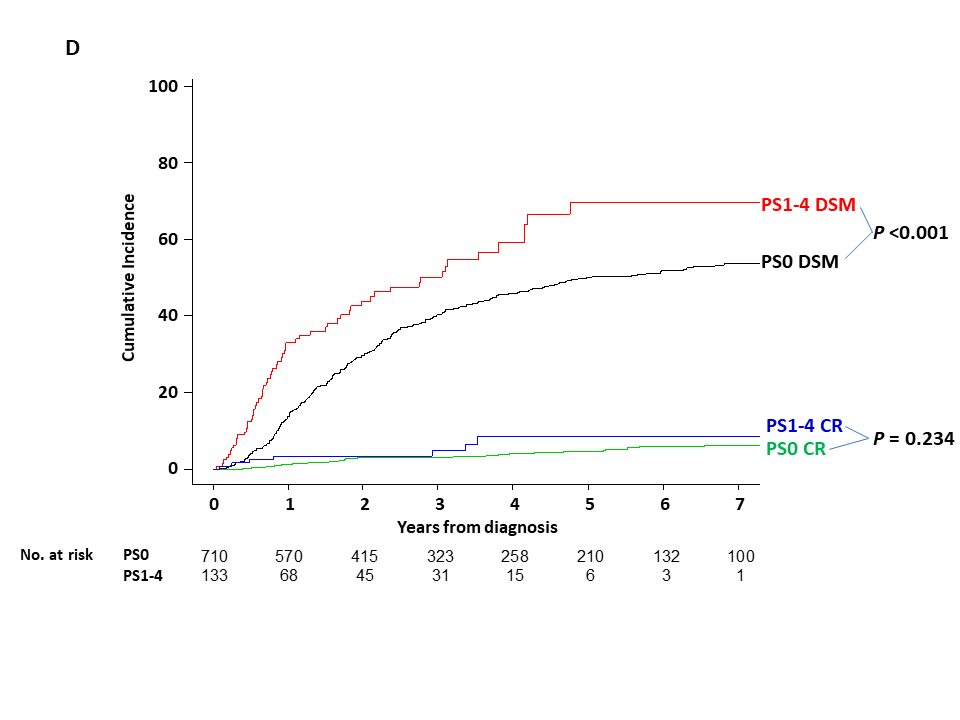


## **FIGURE S6** Survival outcomes in patients with cStage IV disease in the development cohort.

(A) Overall survival stratified by age, (B) Overall survival stratified by the ECOG-PS.


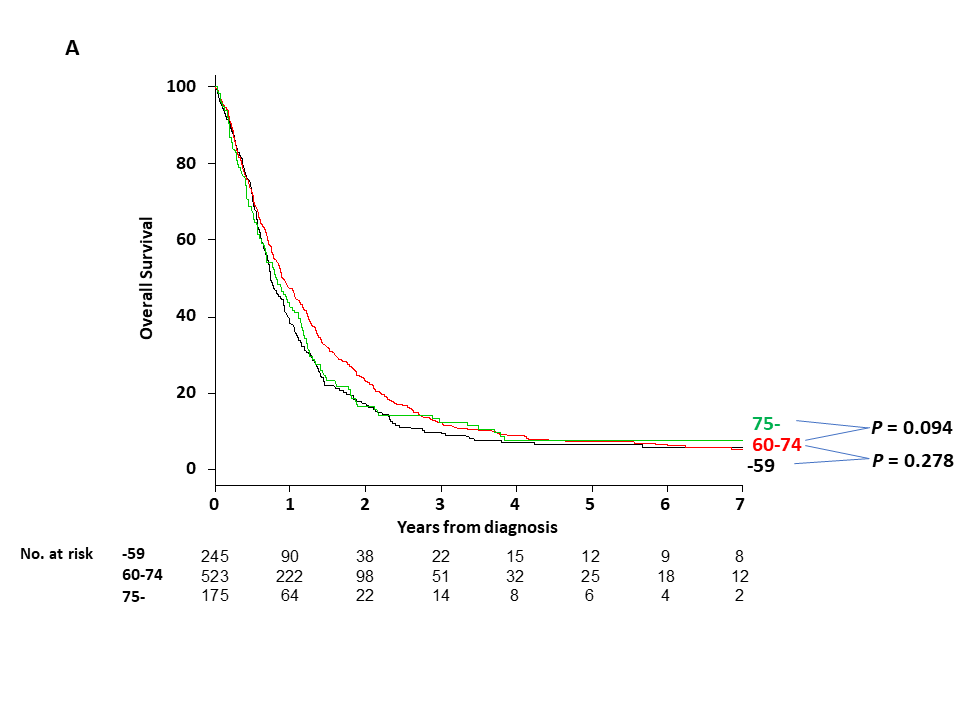

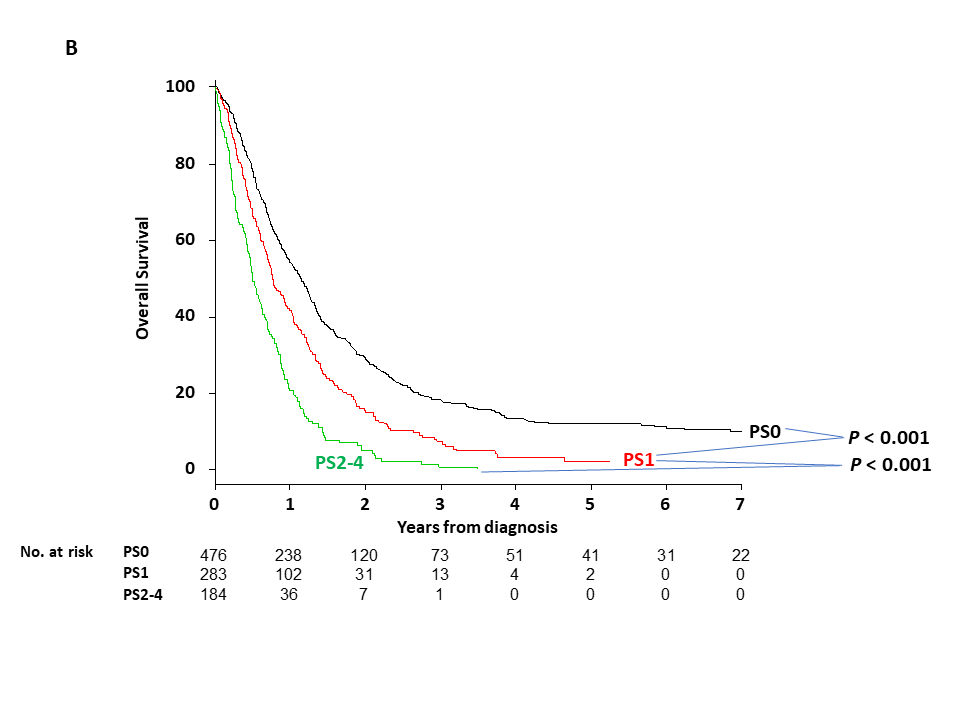


(C) Disease-specific mortality and competing risks stratified by age, (D) Disease-specific mortality and competing risks stratified by the ECOG-PS.


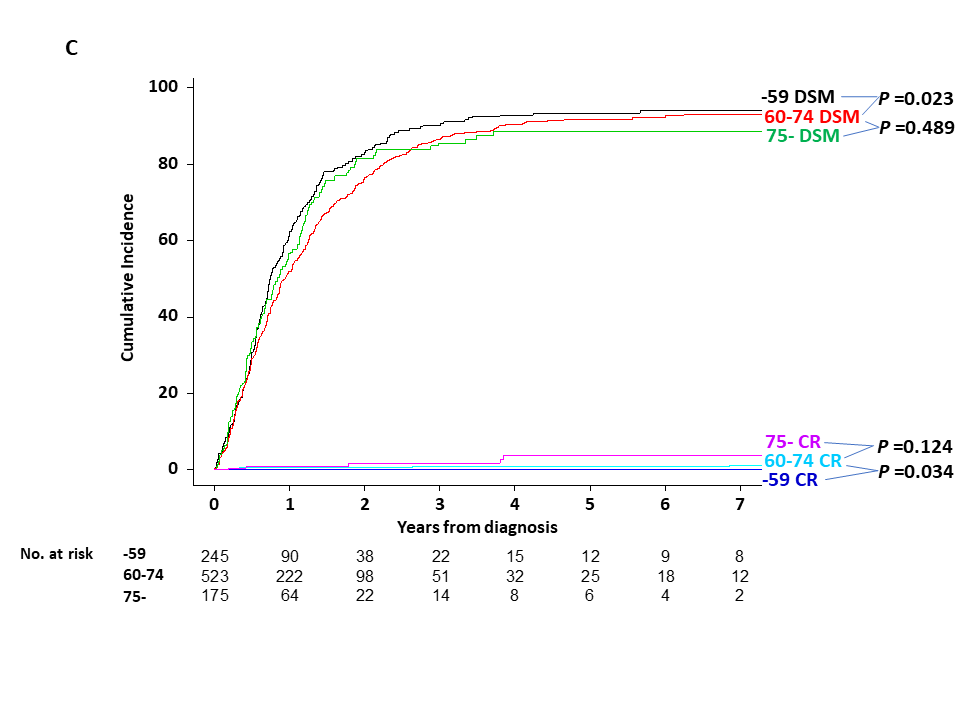

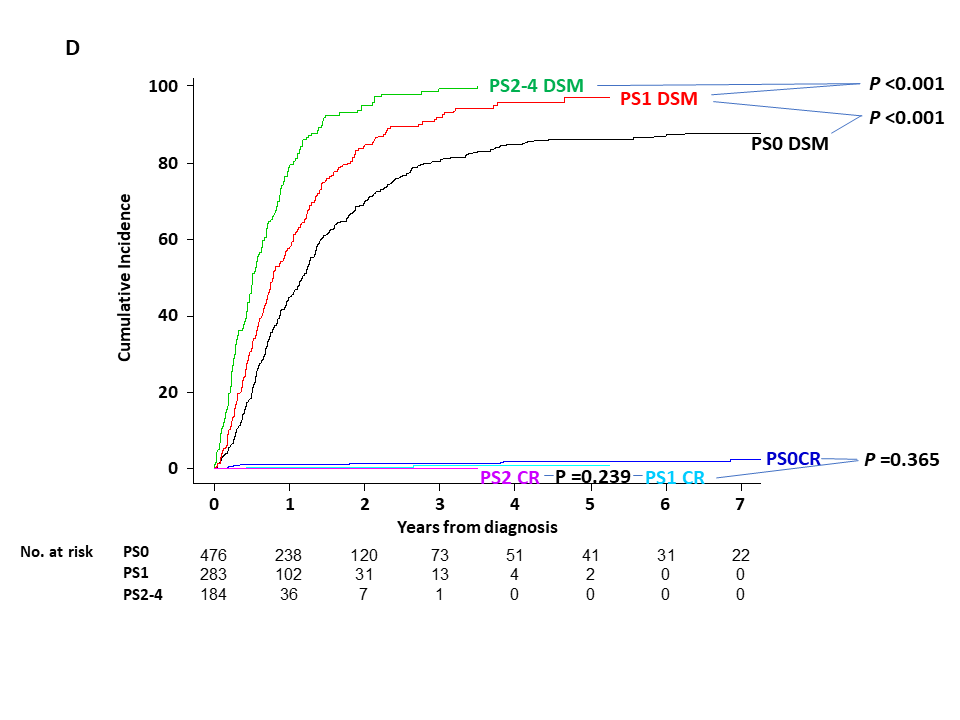


## **FIGURE S7** Survival outcomes in the external validation cohort (all patients).

(A) Overall survival stratified by age, (B) Overall survival stratified by the ECOG-PS.


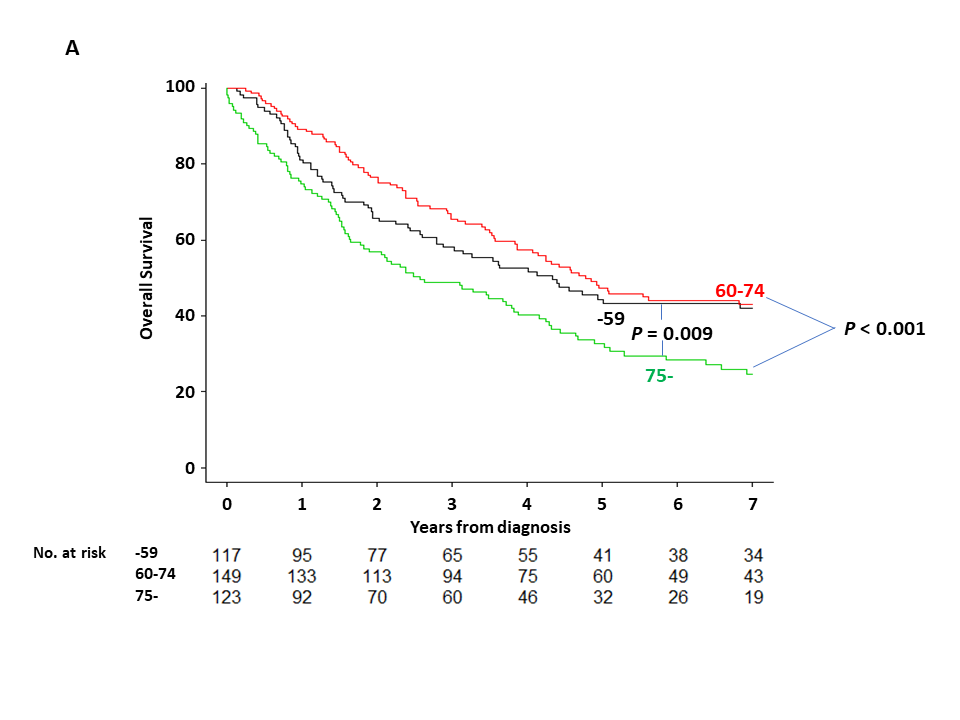

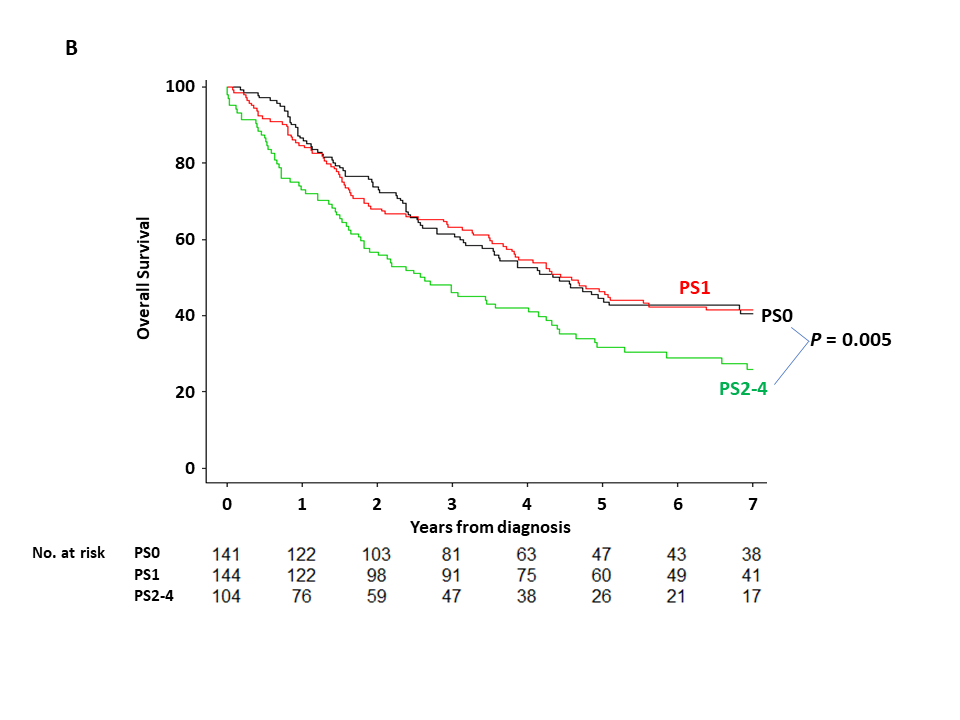


(C) Disease-specific mortality and competing risks stratified by age, (D) Disease-specific mortality and competing risks stratified by the ECOG-PS.


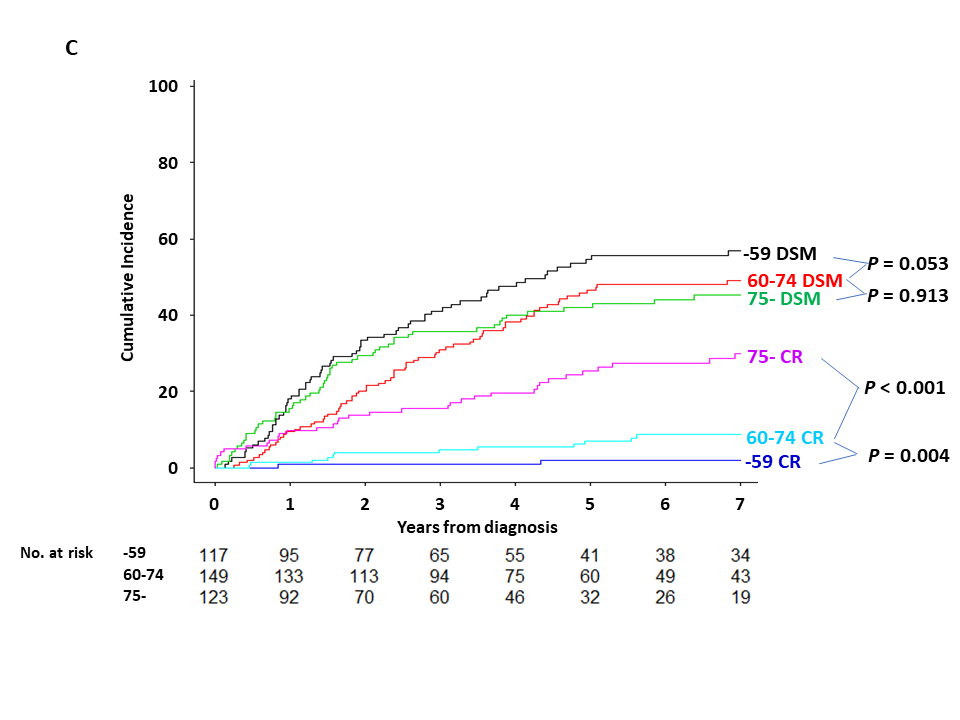

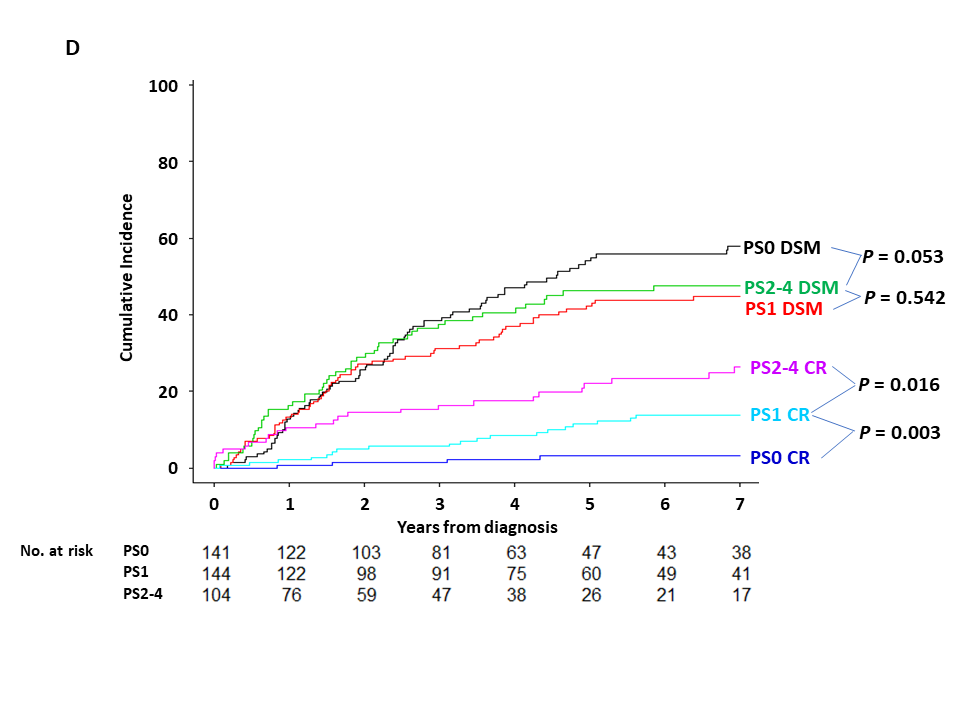


## **FIGURE S8** Survival outcomes in patients with cStage I disease in the external validation cohort.

(A) Overall survival stratified by age, (B) Overall survival stratified by the ECOG-PS.


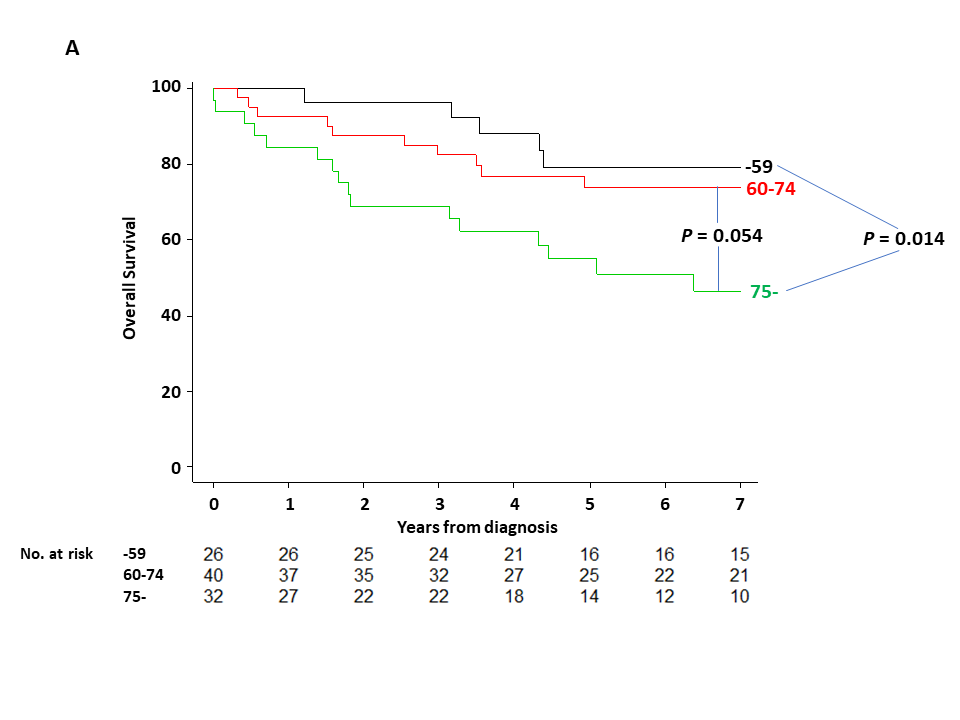


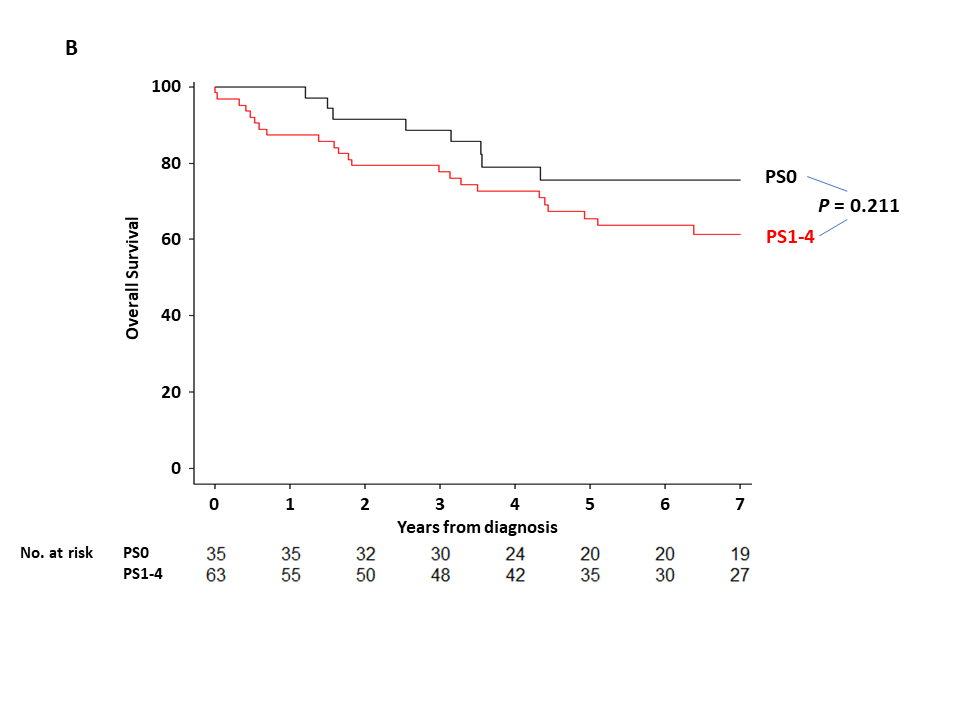


(C) Disease-specific mortality and competing risks stratified by age, (D) Disease-specific mortality and competing risks stratified by the ECOG-PS.


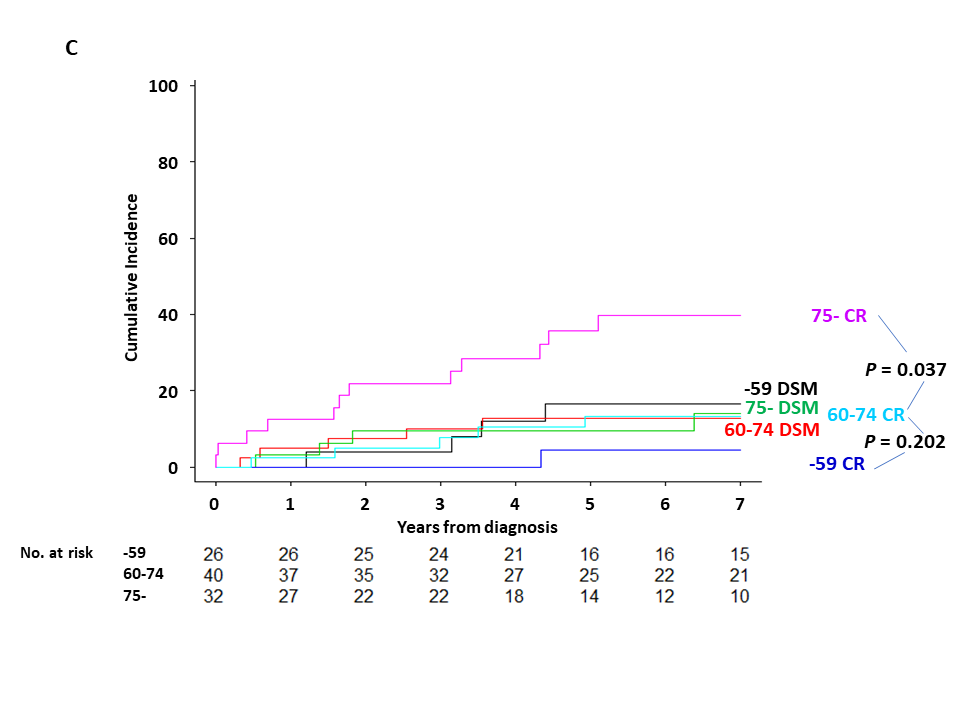

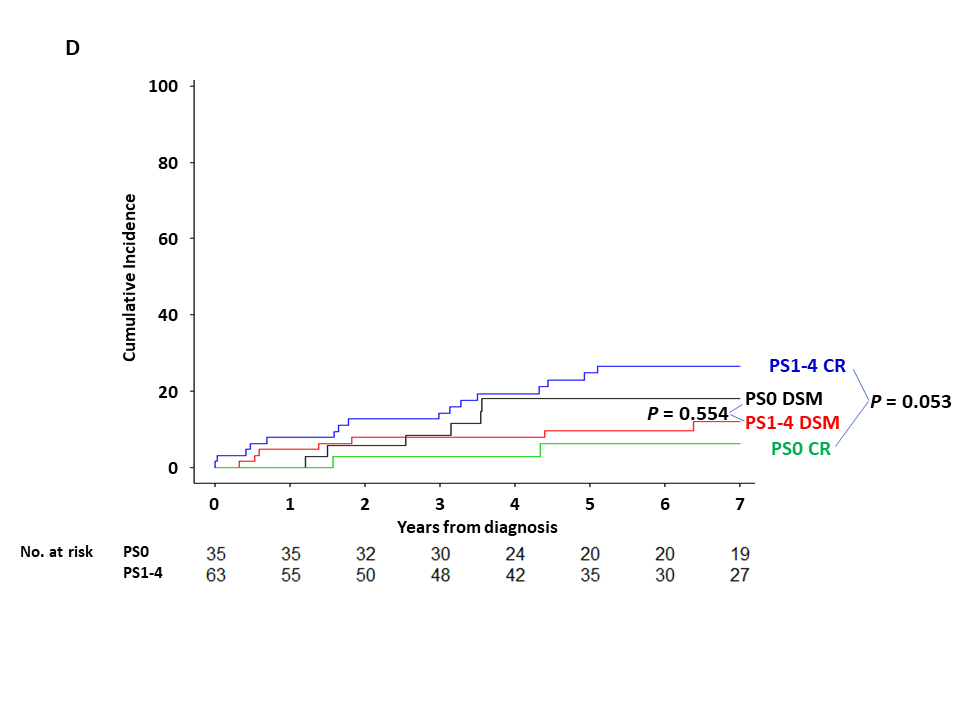


## **FIGURE S9** Survival outcomes in patients with cStage II disease in the external validation cohort.

(A) Overall survival stratified by age, (B) Overall survival stratified by the ECOG-PS.


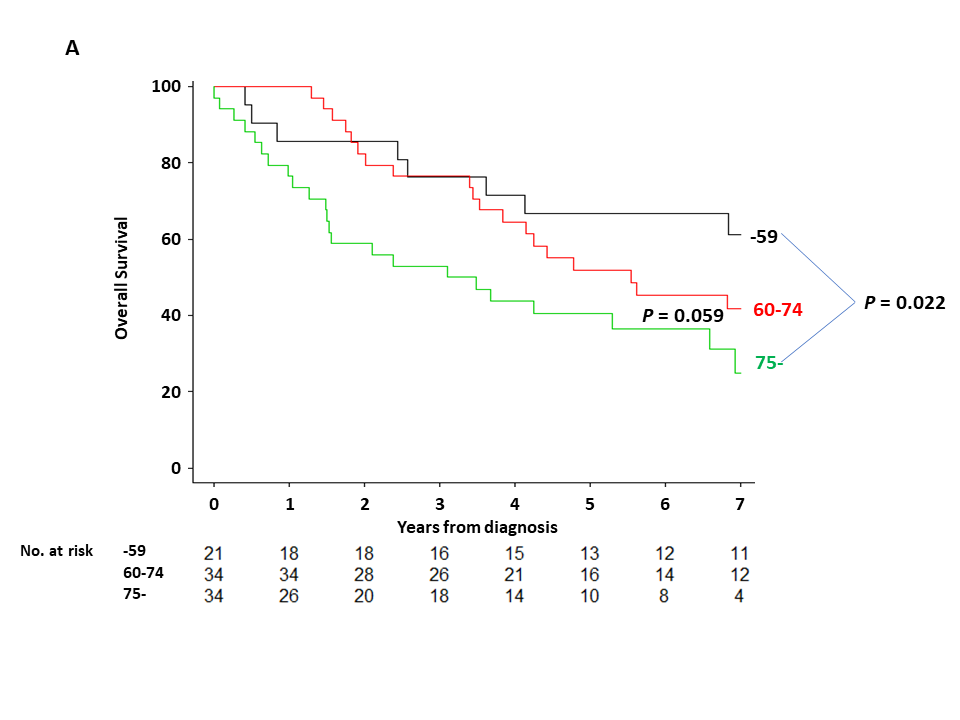

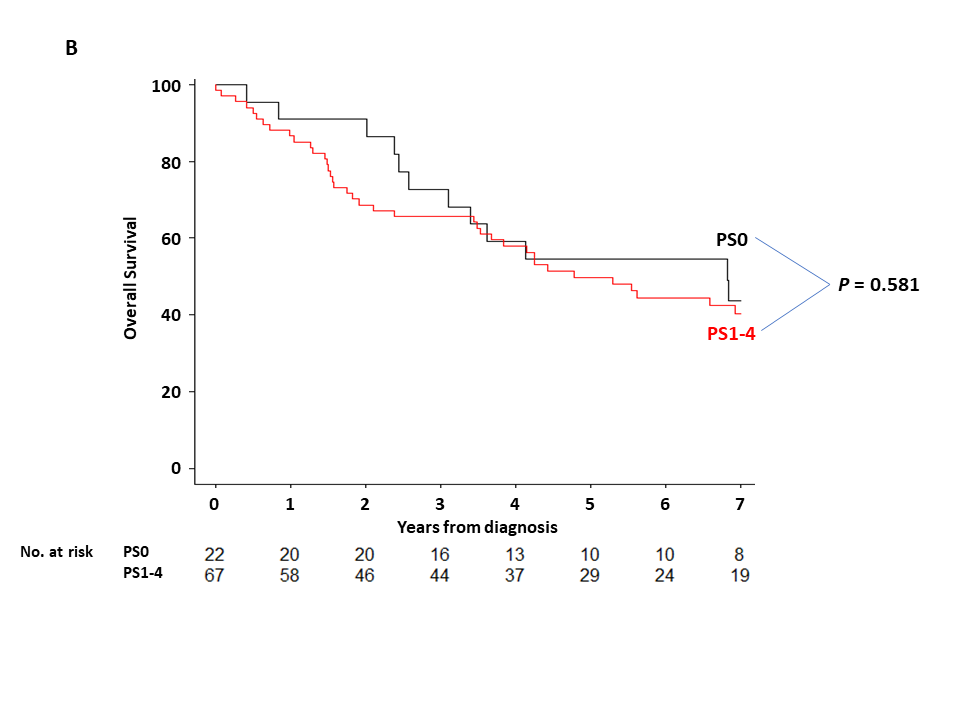


(C) Disease-specific mortality and competing risks stratified by age, (D) Disease-specific mortality and competing risks stratified by the ECOG-PS.


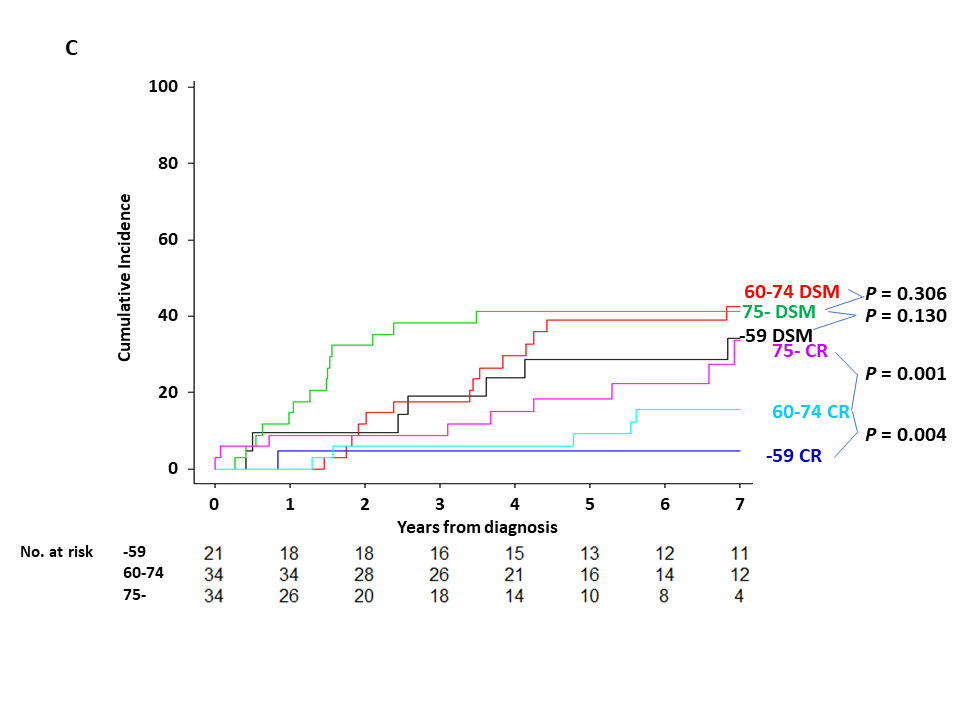

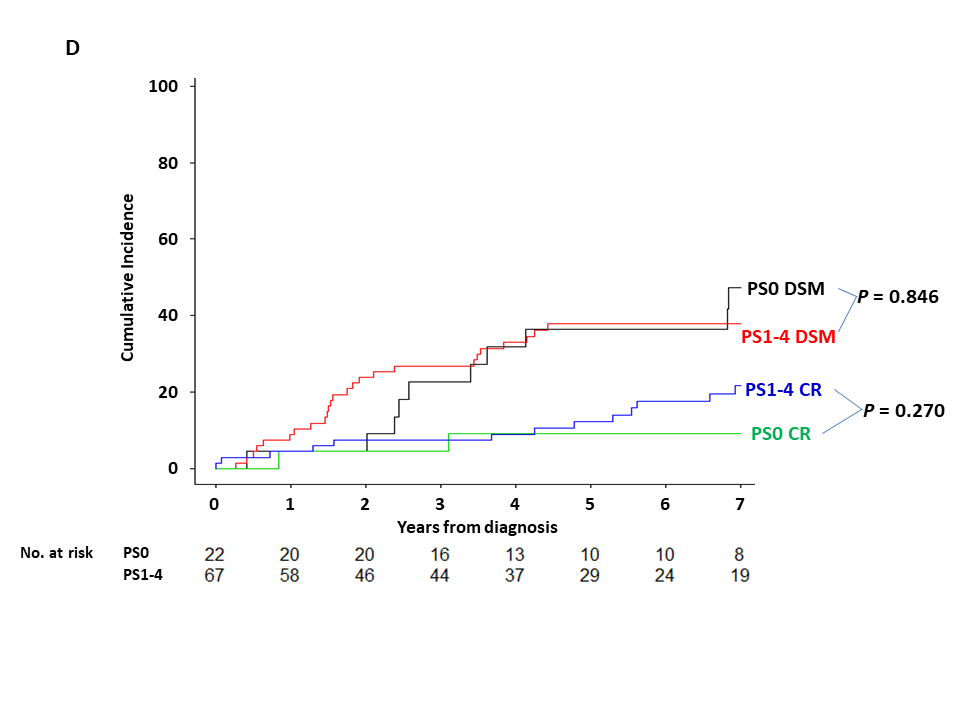


## **FIGURE S10** Survival outcomes in patients with cStage III disease in the external validation cohort.

(A) Overall survival stratified by age, (B) Overall survival stratified by the ECOG-PS.


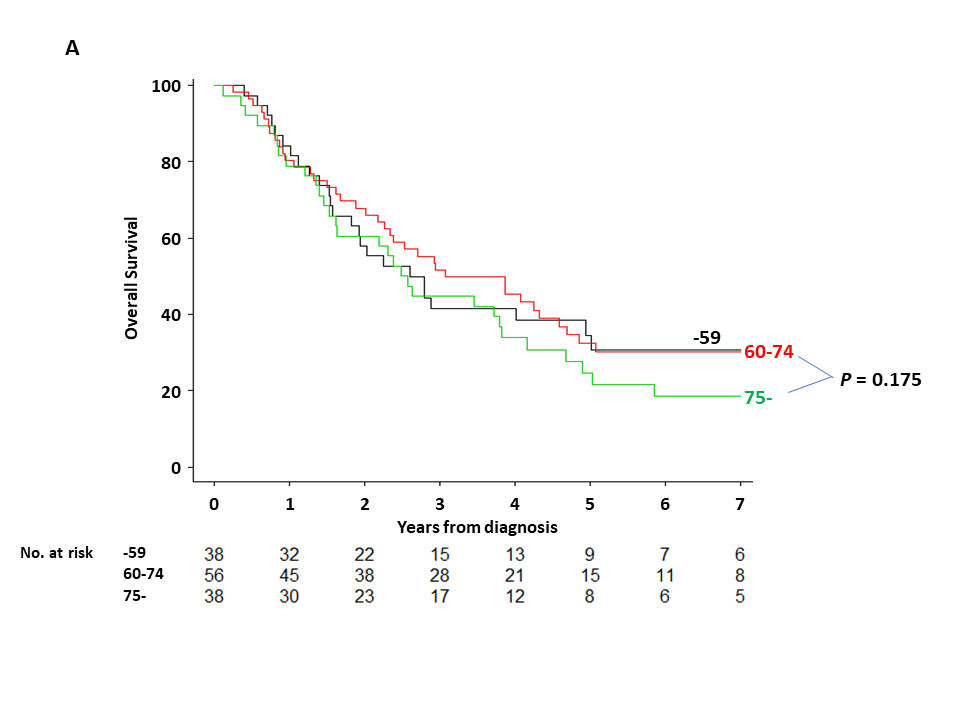

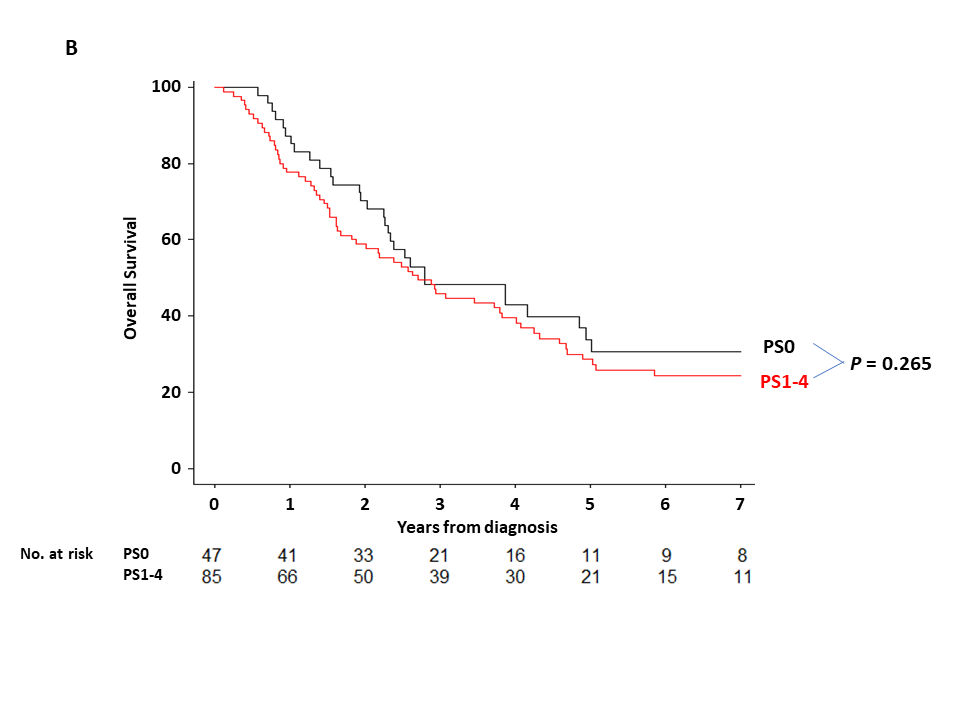


(C) Disease-specific mortality and competing risks stratified by age, (D) Disease-specific mortality and competing risks stratified by the ECOG-PS.


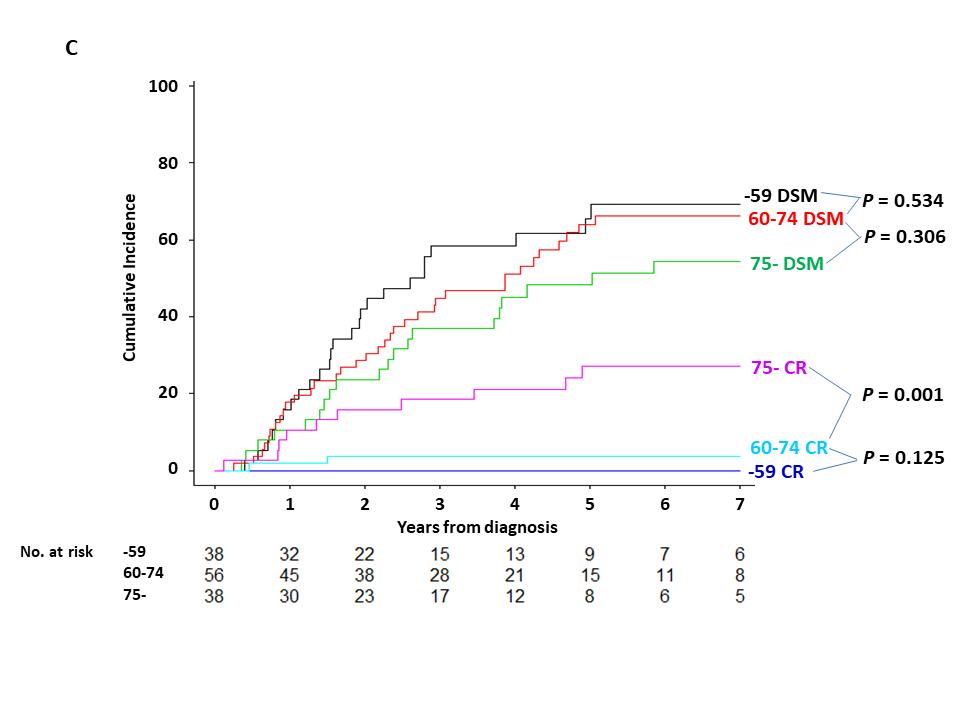

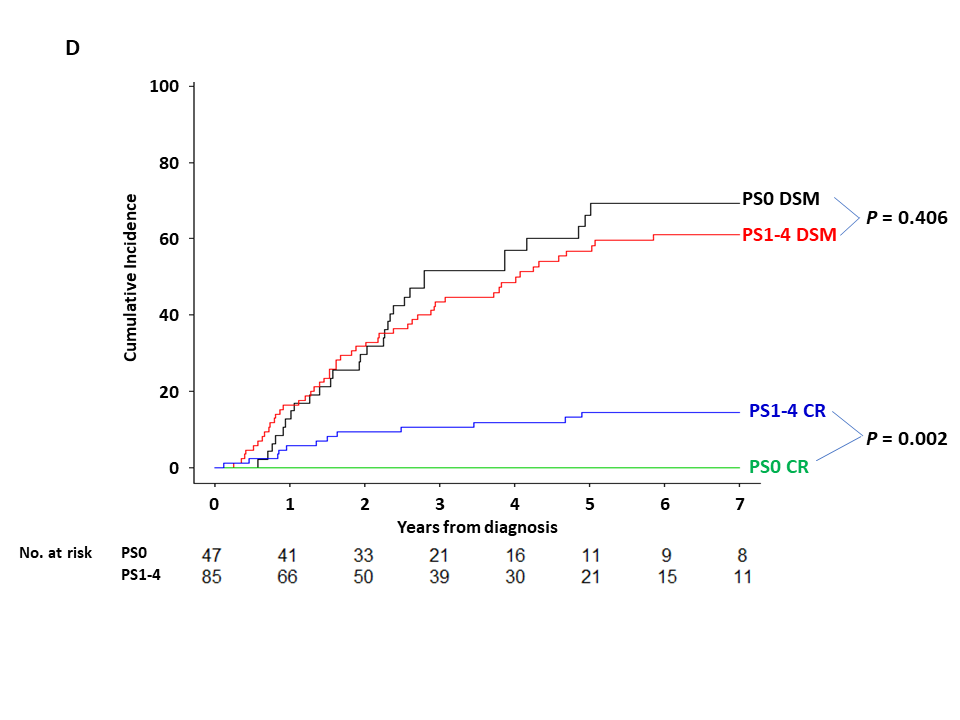


## **FIGURE S11** Survival outcomes in patients with cStage IV disease in the external validation cohort.

(A) Overall survival stratified by age, (B) Overall survival stratified by the ECOG-PS.


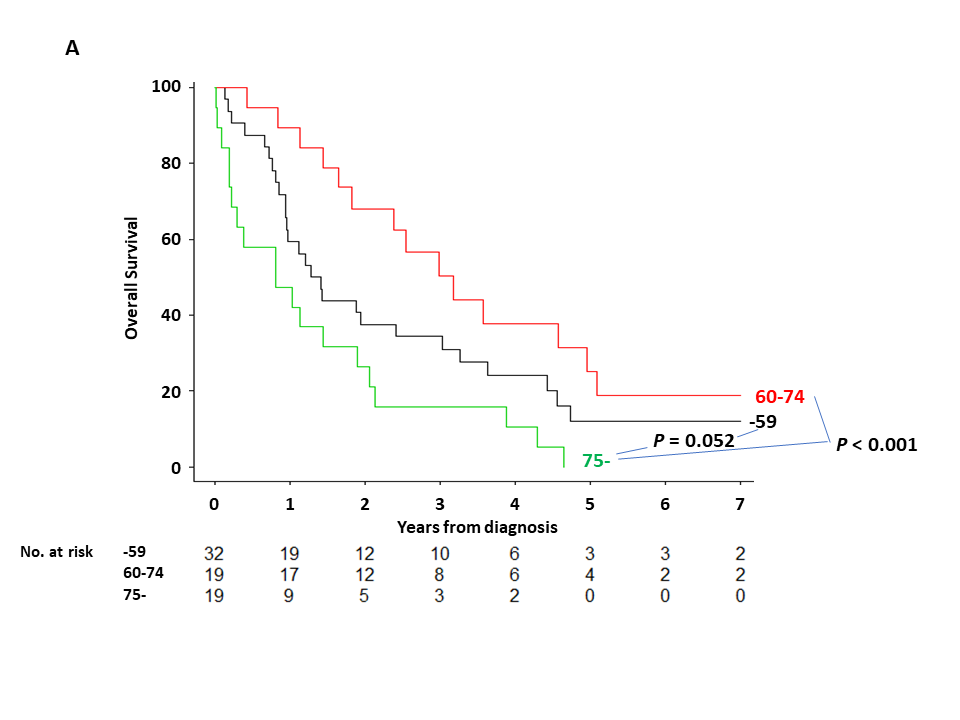

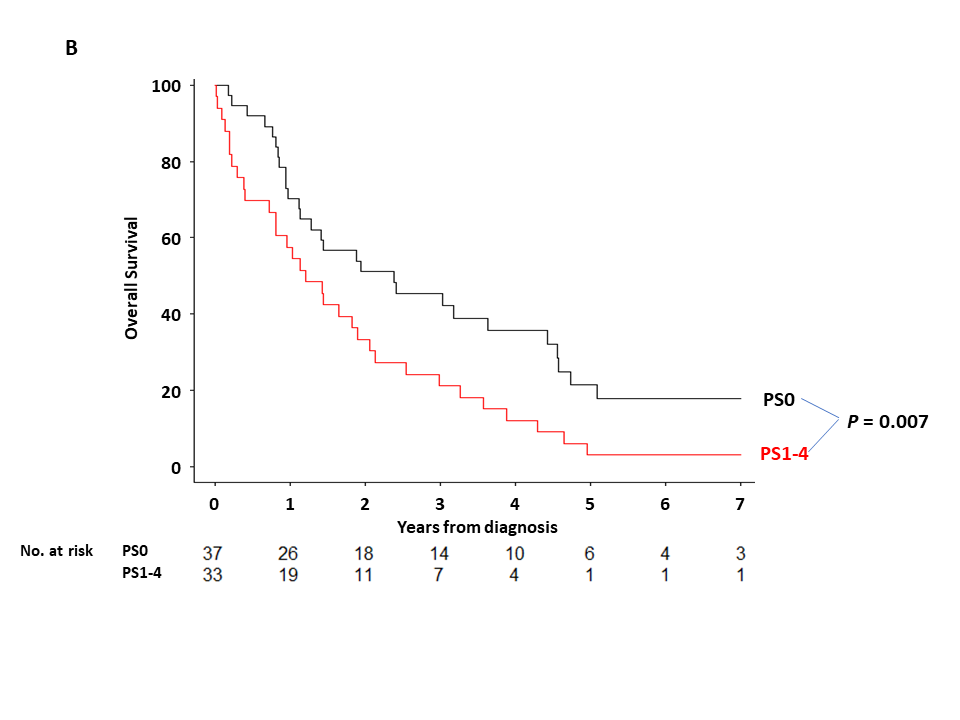


(C) Disease-specific mortality and competing risks stratified by age, (D) Disease-specific mortality and competing risks stratified by the ECOG-PS.


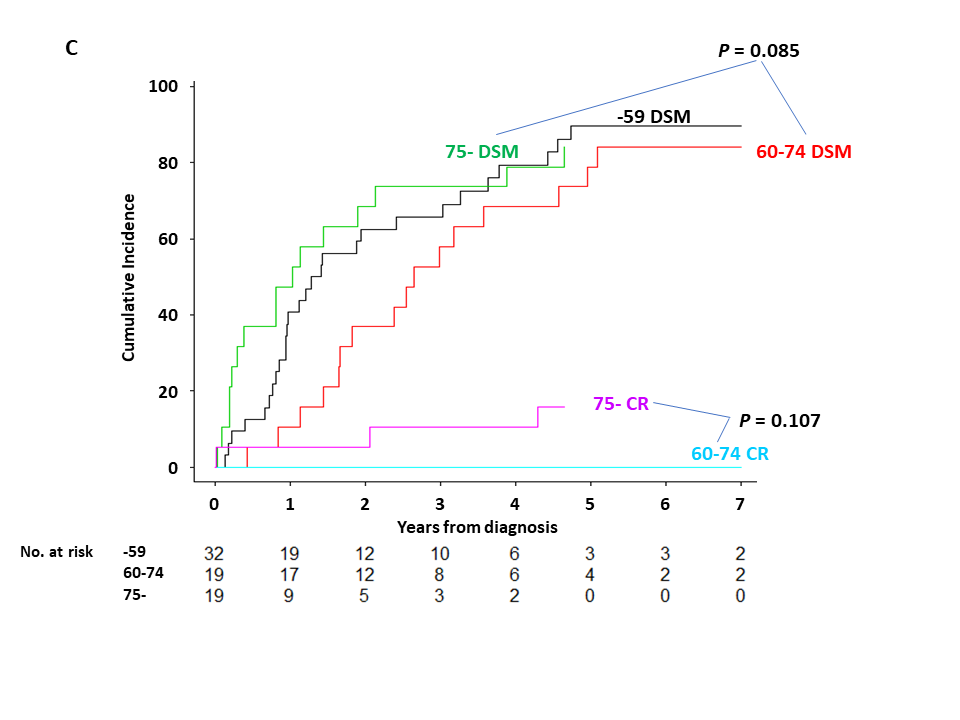

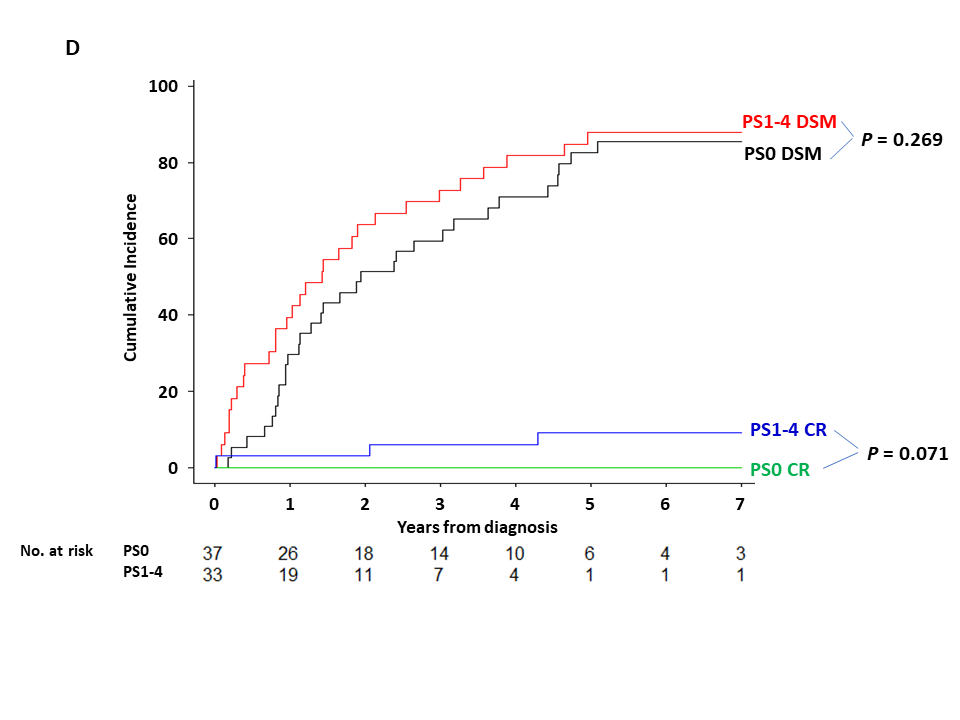


## **FIGURE S12** First example of nomogram use. Patient X: 50-year-old male with cT3 and nodal involvement (American Joint Committee on Cancer stage: cStage III).

(A) Use of the overall survival nomogram. Chance of cure at 5-years was estimated as 27% (overall mortality = 73%).


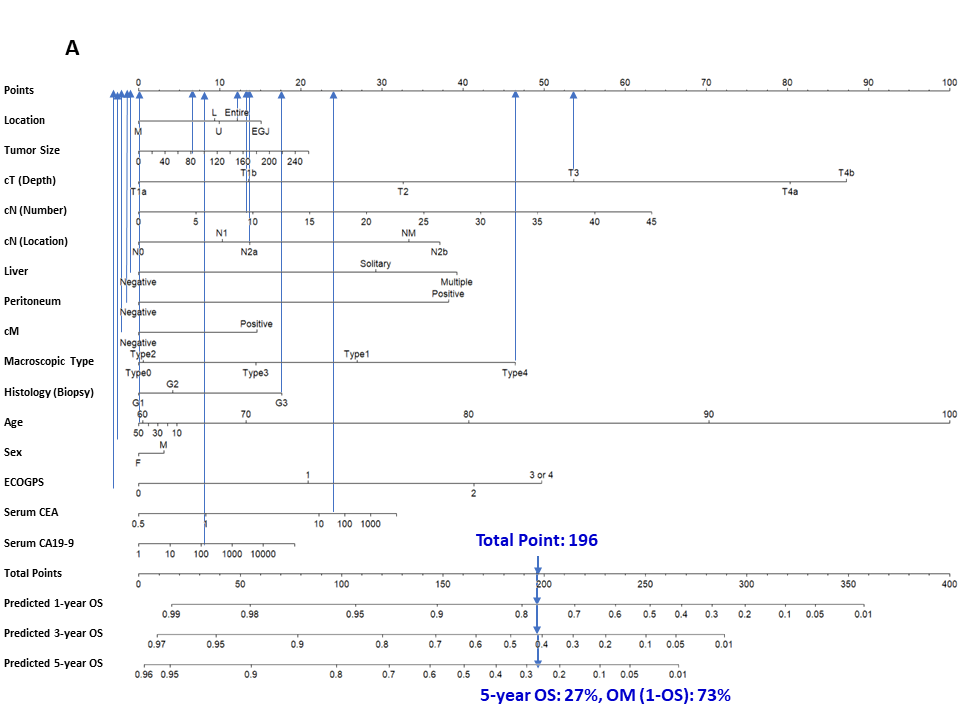


(B) Use of the disease-specific mortality (DSM) nomogram. The cumulative DSM probability at 5-years was estimated as 72%.


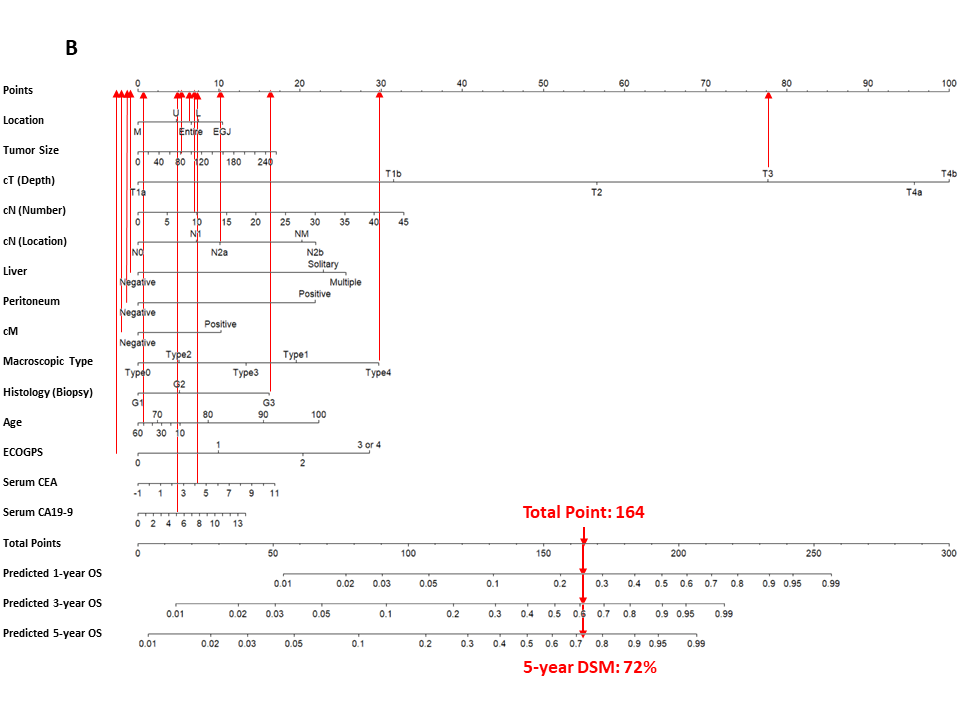


## **FIGURE S13** Second example of nomogram use. Patient Y: 86-year-old male with cT1b but no nodal involvement (American Joint Committee on Cancer stage: cStage I).

(A) Use of the overall survival nomogram. Chance of cure at 5-years was estimated as 60% (overall mortality = 40%).


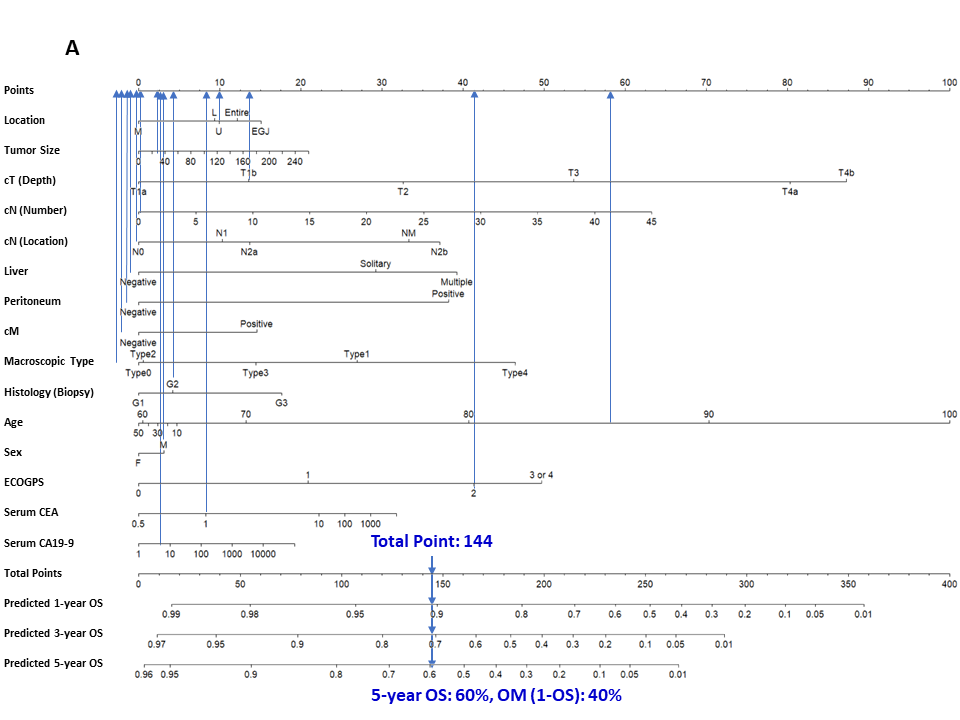


(B) Use of the disease-specific mortality (DSM) nomogram. Cumulative DSM probability at 5-years was estimated as only 9%.


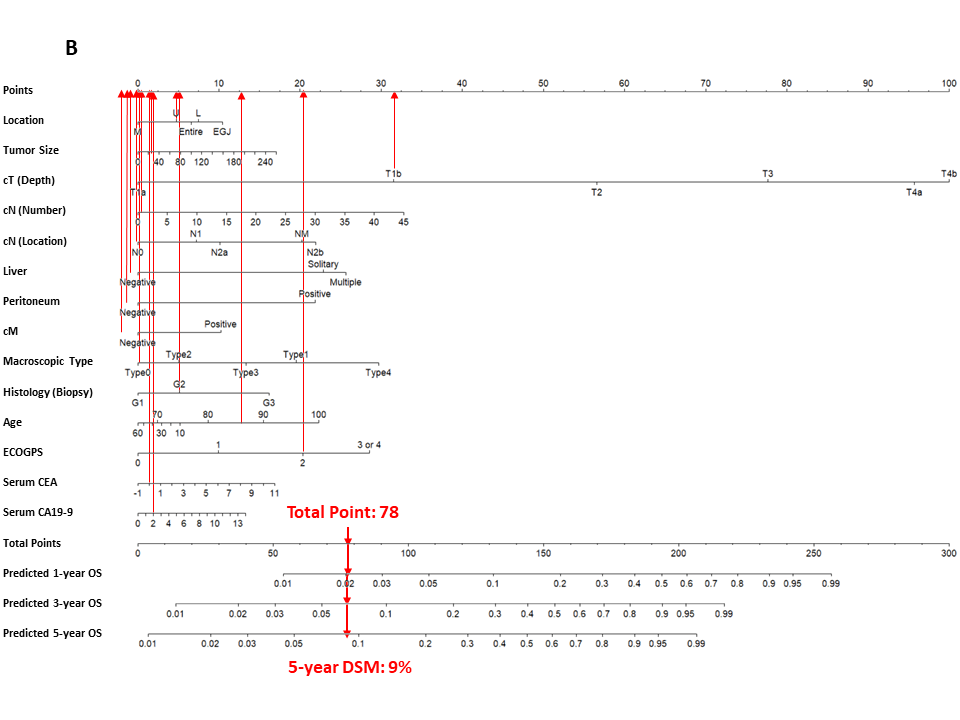


## **FIGURE S14** Practical use of gastric cancer risk calculator.

(A) Calculation of survival outcomes of Patient X.


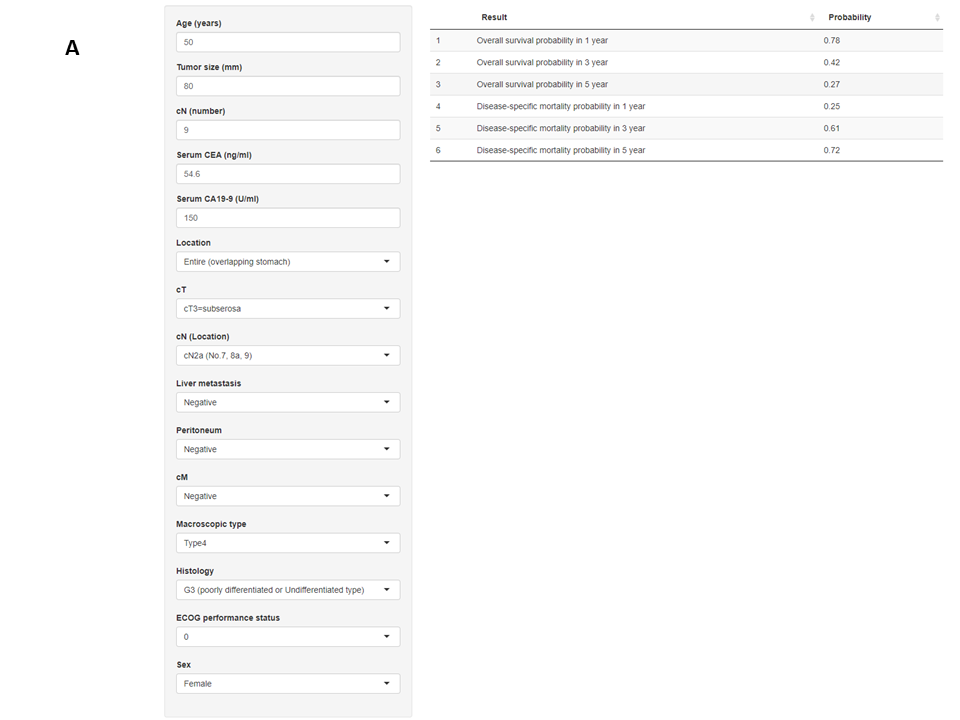


(B) Calculation of survival outcomes of Patient Y.


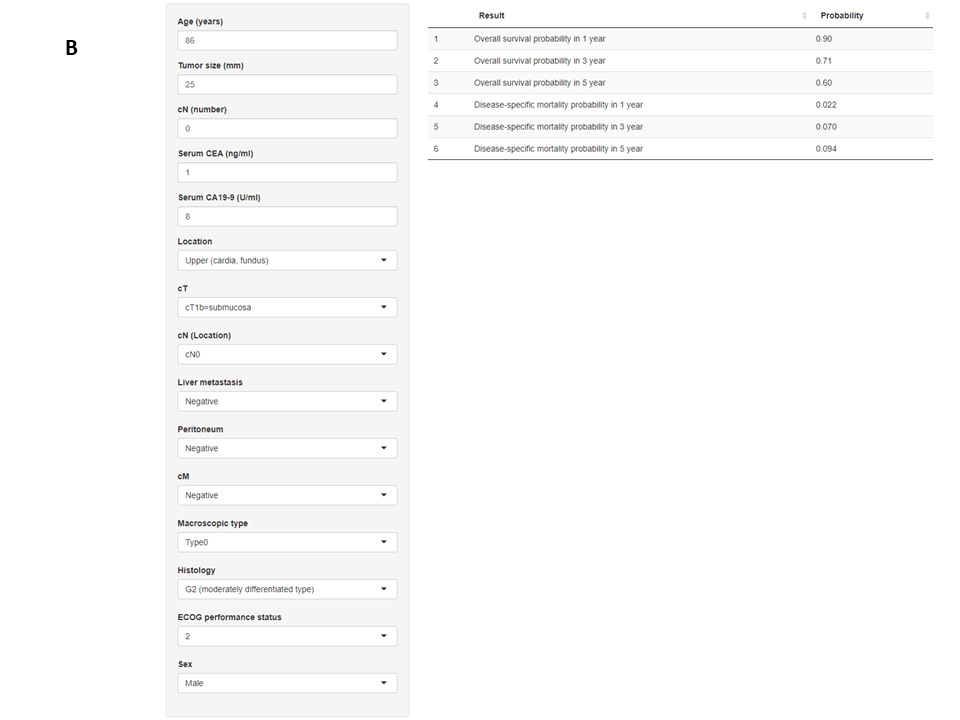

Supplement: Supplementary file 1 — Supplementary Material [file CAM4-10-7561-s001.docx]
